# Oral Frailty, Dental Visits, and Healthy Life Expectancy: A 6‐Year Prospective Cohort Among Japanese Older Adults

**DOI:** 10.1111/ggi.70230

**Published:** 2025-10-21

**Authors:** Safira Khairinisa, Sakura Kiuchi, Yusuke Matsuyama, Masanori Iwasaki, Jun Aida

**Affiliations:** ^1^ Department of Dental Public Health, Graduate School of Medical and Dental Sciences Institute of Science Tokyo Tokyo Japan; ^2^ Department of Preventive Dentistry, Faculty of Dental Medicine and Graduate School of Dental Medicine Hokkaido University Sapporo Japan

**Keywords:** cohort study, dental visit, geriatric dentistry, healthy life expectancy, oral frailty

## Abstract

**Aim:**

Oral frailty (OF), a comprehensive oral function decline, is linked to health deterioration in older adults. The impact of OF and dental visits on healthy life expectancy (HLE) remains underexplored. This study examined the association between OF, dental visits, and HLE.

**Methods:**

This prospective cohort study used data from the 2016 Japan Gerontological Evaluation Study (JAGES), including independent older adults aged ≥ 65 at baseline, with follow‐up through March 2023 using disability and mortality records from the national long‐term care insurance database (median follow‐up = 6.2 years). OF was defined as having ≥ 3 of the following: fewer teeth, chewing difficulty, swallowing difficulty, dry mouth, and speaking difficulty. Recent dental visits at baseline were assessed. HLE by OF status and dental visits was estimated using Royston–Parmar multistate modeling in random forest‐imputed datasets, adjusting for sociodemographic and health confounders.

**Results:**

Among 11 080 participants (mean age 74.1; 52.9% female), 12.0% were OF, and 49.5% had a dental visit in the past 6 months. OF was associated with transition from healthy to disability (hazard ratio [HR]: 1.23, 95% confidence interval [95% CI]: 1.01–1.50) and from healthy to death (HR: 1.34, 95% CI: 1.05–1.71). At age 65, males without OF had an HLE of 23.39 years, while males with OF had 21.96 years; females without OF had an HLE of 24.77 years, while females with OF had 23.64 years. Dental visits were associated with approximately 1 year longer HLE in all groups.

**Conclusion:**

OF significantly reduces HLE, and dental visits may mitigate this outcome in older adults.

## 1 Introduction

In an aging world, while global life expectancy (LE) has steadily increased over the past few decades, many older adults face prolonged periods of functional decline, chronic health conditions, and reduced quality of life [1]. Japan is a “super aging” country with one of the longest LEs in the world. In 2023, the average LE is 81.09 years for males and 87.14 years for females [2]. The focus has shifted from merely extending LE to increasing healthy life expectancy (HLE), the number of years a person lives in good health, free from major disability [3].

Research shows that maintaining good oral health, including retaining natural teeth and preserving oral function, helps older people live longer, healthier lives with less need for long‐term care [4, 5, 6]. While tooth loss has traditionally been a key indicator of oral health in older adults, it is irreversible and does not provide a complete picture of oral health status [5, 7]. Oral frailty (OF) provides a more comprehensive indicator of oral function by including chewing, swallowing, and speaking, which may have a greater impact on HLE [8, 9]. It refers to the decline in oral health and function associated with aging, which contributes significantly to physical decline, mental deterioration, and reduced quality of life [7, 10].

While previous studies have examined the association between tooth loss and LE [5, 11, 12, 13], few have specifically examined OF as a comprehensive domain in estimating HLE. Given that effective prevention and treatment of OF require multidisciplinary approaches, dental care plays a central role in preserving oral function and preventing further complications in older adults [5, 7, 14, 15]. This study examines the association between OF and dental visits with HLE among community‐dwelling older adults in Japan.

## 2 Methods

The Japan Gerontological Evaluation Study (JAGES) is an ongoing cohort study investigating social, environmental, and behavioral determinants of health decline, particularly functional decline and cognitive impairment, among community‐dwelling adults aged 65 years or older [16]. This prospective cohort study analyzed data from the 2016 JAGES survey in 21 municipalities, including participants who had not been certified as requiring long‐term care under Long‐term Care Insurance (LTCI). They were followed for approximately 6 years, from 2016 (baseline) to March 2023 (median follow‐up = 6.2 years) [16].

In nine larger municipalities, a random sampling approach was used, while in the other 12 smaller municipalities, all eligible residents were invited using local registries. A total of 144 046 questionnaires were distributed. Of the 95 070 respondents (66.0% response rate), data from 93 406 were available for follow‐up (98.2% follow‐up rate). The survey included core items and thematic items, with eight different versions of the latter. This study focused on respondents who completed version A, which included oral health questions (*n* = 11 687). Missing data were imputed, resulting in a final analyzed sample of 11 080 respondents, after excluding 607 who reported needing nursing care or assistance in daily life at baseline. The flowchart of study participants is shown in Figure S1.

## 3 Exposure

OF and dental visits were the exposures of this study. OF was assessed using the JAGES 2016 survey items, similar to the OF‐5 defined by three academic societies in Japan, which includes five components [7]. While four of the five OF‐5 questions regarding fewer teeth (0 = more than 20 teeth; 1 = 0–19 teeth), chewing difficulty (0 = no; 1 = yes), swallowing difficulty (0 = no; 1 = yes), and dry mouth (0 = no; 1 = yes) matched those from the JAGES 2016, one question regarding low articulatory oral motor skills was replaced with a comparable item in the current assessment, trouble pronouncing clearly in the last 6 months (0 = no; 1 = yes) [7, 9, 13]. Different from the latest consensus, where OF from OF‐5 is classified in a two‐category [7], this study classified participants into three OF levels to determine a dose–response association: non‐OF (score 0), pre‐OF (score 1–2), and OF (score 3–5), which was based on a previous study that showed a dose–response association between OF and disability or death [9]. In one of the sensitivity analyses, we also used a two‐category classification of OF based on the latest consensus [7].

Dental visits were based on whether respondents had visited a dentist in the past 6 months, including both routine and treatment visits. Participants were classified as having had a dental visit if either indicator was answered “yes,” and as having had no dental visits if both indicators were answered “no.”

## 4 Outcome

The primary outcome of the study was HLE, measured from baseline to the onset of disability or mortality. Disability was defined as care level 2 or higher in Japan's LTCI system, indicating significant limitations in activities of daily living (ADL) [5, 12, 17]. Mortality data were also obtained from LTCI records. Following previous studies, transitions were analyzed to model the progression from a healthy state to disability (Transition 1), from a healthy state to death (Transition 2), and from a disabled state to death (Transition 3), based on the time of onset and days of survival [18, 19].

## 5 Covariates

Several confounders were included in this study to control for factors that may influence LE [5, 11, 17, 19]. These included age (65–69, 70–74, 75–79, 80–84, and 85+), sex, marital status (married/other), living arrangement (alone/with others), education level (≤ 9 years/> 9 years), employment status (employed/unemployed), and household income (< 2, 2–4, > 4 million JPY). Body mass index (BMI) was calculated from self‐reported weight and height, and was categorized as underweight (< 18.5 kg/m^2^), normal weight (18.5–24.9 kg/m^2^), and overweight/obese (≥ 25 kg/m^2^). Health conditions (hypertension, diabetes, stroke, cancer, dementia) were binary (presence/absence). Depressive symptoms were assessed using the Geriatric Depression Scale (GDS)‐15, and were classified as no depression (0–4), suggested depression (5–9), and depression (10–15) [20, 21]. Smoking and alcohol status were categorized as a non‐smoker, past smoker, or current smoker; and non‐drinker, past drinker, or current drinker. Walking time was used as a proxy for exercise, categorized as ≥ 30 min or < 30 min per day.

### 5.1 Statistical Analysis

Descriptive statistics were used to assess the distribution of OF and dental visit status across covariates. Proportional hazards were evaluated with the Schoenfeld test in Cox estimates for each transition (*p* > 0.05) [22]. A multistate survival model with the Royston–Parmar flexible parametric approach estimated transition probabilities among three states (Figure S2) for different OF and dental visit statuses. The use of flexible parametric modeling ensures that the baseline hazard is accurately captured and provides sufficient flexibility to accommodate potential changes in risk over time by using a restricted cubic spline [23]. The onset of transitions was examined, and hazard ratios (HRs) with 95% confidence intervals (CIs) were calculated for each transition. Transition probabilities were tracked until they reached an absorbing state (death) or were censored (e.g., maximum follow‐up, relocation). These probabilities were further used to estimate HLE, life expectancy in disability (LED), and total life expectancy (TLE).

The analysis was performed using Stata's “multistate” and “stmerlin” packages. Three models were developed to assess the impact of OF on transition probabilities in different adjustment models: Model I, which was adjusted for age and sex; Model II, which included dental visits; and Model III, which included all covariates. HLE, LED, and TLE at 65 years old for males and females across various states of OF and dental visit status were estimated using the “predictms” command based on the fully adjusted model (Model III).

The prediction model assigned the observed categorical values to primary variables (sex, age, OF status, and dental visits), and other confounders were assigned their prevalence to estimate population‐average outcomes. Estimates from the multistate models with 6‐year follow‐up data were extended by assuming that the association would continue for 30 years, following the previous study's approach [11]. The analyses were performed using Stata 17.0 (Stata Corporation LP, Windows version) [24, 25]. Random forest imputation with the R package “missForest” in R 4.4.1 was used to handle missing data. The significance level was set at *p* < 0.05.

To validate the robustness of our findings, sensitivity analyses excluding individuals who developed disability or died within 1 year of baseline were conducted (*n* = 10 918). Additionally, the final analysis (*n* = 11 080) was performed using the Weibull and Gompertz distributions instead of the Royston‐Parmar model. Results for complete case analysis before imputation (*n* = 5860; Figure S3) and sensitivity analysis with multiple cut‐offs of OF classifications were also presented.

### 5.2 Ethical Issues

The JAGES 2016 study followed ethical guidelines approved by the ethics committees of the National Center for Geriatrics and Gerontology (Approval No. 992), Chiba University (Approval No. 2493), and the Institute of Science Tokyo (D2022‐040‐01). Participants received a written explanation and completed the questionnaire if they agreed to participate.

## 6 Result

Among 11 080 participants (mean age 74.1, 52.9% female), 33.9% were non‐OF, 54.1% were pre‐OF, 12.0% were OF, and 49.5% had a dental visit in the past 6 months. Participant characteristics by OF status and dental visits are shown in Table 1, with additional details in Tables S1–S3. Table 2 shows the incident of disability or death in each transition of different OF and dental visit status, highlighting a progressive onset for those with worse OF status and no dental visit, especially in transition 1 (healthy to disabled) and transition 2 (healthy to death). In all groups, those with a dental visit within the last 6 months tended to have a lower risk of disability and death, with larger differences observed in those with worse OF status.

**TABLE 1:** Baseline demographic and characteristic based on oral frailty status and dental visit after imputation (*n* = 11 080).

	Overall		Oral frailty status						Dental visit in the last 6 months			
			Non‐OF (0)		Pre‐OF (1, 2)		OF (3–5)		No visit		Visit	
	*n* = 11 080	%col	*n*	% row	*n*	% row	*n*	%row	*n*	% row	*n*	%row
			3756	33.9	5997	54.1	1327	12.0	5594	50.5	5486	49.5
Age												
65–69	3218	29.0	1411	43.8	1530	47.5	277	8.6	1568	48.7	1650	51.3
70–74	3082	27.8	1086	35.2	1686	54.7	310	10.1	1682	54.6	1400	45.4
75–79	2586	23.3	821	31.7	1410	54.5	355	13.7	1393	53.9	1193	46.1
80–84	1505	13.6	337	22.4	928	61.7	240	15.9	704	46.8	801	53.2
85+	689	6.2	101	14.7	443	64.3	145	21	247	35.8	442	64.2
Sex												
Female	5858	52.9	2022	34.5	3202	54.7	634	10.8	3089	52.7	2769	47.3
Male	5222	47.1	1734	33.2	2795	53.5	693	13.3	2505	48	2717	52
Marital status												
Married	8176	73.8	2994	36.6	4294	52.5	888	10.9	4234	51.8	3942	48.2
Not married	2904	26.2	762	26.2	1703	58.6	439	15.1	1360	46.8	1544	53.2
Living arrangement												
Living alone	1688	15.2	467	27.7	983	58.2	238	14.1	810	48	878	52
Not living alone	9392	84.8	3289	35	5014	53.4	1089	11.6	4784	50.9	4608	49.1
Educational attainment												
≤ 9 years	3647	32.9	927	25.4	2137	58.6	583	16	1583	43.4	2064	56.6
> 9 years	7433	67.1	2829	38.1	3860	51.9	744	10	4011	54	3422	46
Employment												
Employed	2927	26.4	1083	37	1541	52.6	303	10.4	1421	48.5	1506	51.5
Not employed	8153	73.6	2673	32.8	4456	54.7	1024	12.6	4173	51.2	3980	48.8
Equivalent income												
< 2 million JPY/year	2901	26.2	697	24	1668	57.5	536	18.5	1214	41.8	1687	58.2
2–4 million JPY/year	4607	41.6	1658	36	2479	53.8	470	10.2	2418	52.5	2189	47.5
> 4 million JPY/year	3572	32.2	1401	39.2	1850	51.8	321	9	1962	54.9	1610	45.1
BMI												
Under weight	785	7.1	227	28.9	427	54.4	131	16.7	398	50.7	387	49.3
Normal weight	7594	68.5	2657	35	4061	53.5	876	11.5	3931	51.8	3663	48.2
Over weight	2701	24.4	872	32.3	1509	55.9	320	11.8	1265	46.8	1436	53.2
GDS‐15												
No (0–4)	8939	80.7	3315	37.1	4839	54.1	785	8.8	4617	51.7	4322	48.3
Suggested depression (5–9)	1758	15.9	383	21.8	955	54.3	420	23.9	809	46	949	54
Depression (10–15)	383	3.5	58	15.1	203	53	122	31.9	168	43.9	215	56.1
Dementia												
No	11 051	99.7	3747	33.9	5985	54.2	1319	11.9	5578	50.5	5473	49.5
Yes	29	0.3	9	31	12	41.4	8	27.6	16	55.2	13	44.8
Hypertension												
No	6116	55.2	2169	35.5	3232	52.8	715	11.7	3141	51.4	2975	48.6
Yes	4964	44.8	1587	32	2765	55.7	612	12.3	2453	49.4	2511	50.6
Diabetes												
No	9700	87.5	3351	34.5	5190	53.5	1159	11.9	4914	50.7	4786	49.3
Yes	1380	12.5	405	29.3	807	58.5	168	12.2	680	49.3	700	50.7
Stroke												
No	10 805	97.5	3675	34	5842	54.1	1288	11.9	5451	50.4	5354	49.6
Yes	275	2.5	81	29.5	155	56.4	39	14.2	143	52	132	48
Cancer												
No	10 656	96.2	3641	34.2	5744	53.9	1271	11.9	5358	50.3	5298	49.7
Yes	424	3.8	115	27.1	253	59.7	56	13.2	236	55.7	188	44.3
Smoking												
Non‐smoker	6649	60.0	2456	36.9	3510	52.8	683	10.3	3527	53	3122	47
Past smoker	3274	29.5	1034	31.6	1801	55	439	13.4	1625	49.6	1649	50.4
Current smoker	1157	10.4	266	23	686	59.3	205	17.7	442	38.2	715	61.8
Drinking												
Non‐drinker	5654	51.0	1870	33.1	3135	55.4	649	11.5	2896	51.2	2758	48.8
Past drinker	1177	10.6	304	25.8	671	57	202	17.2	533	45.3	644	54.7
Current drinker	4249	38.3	1582	37.2	2191	51.6	476	11.2	2165	51	2084	49
Walking time												
≥ 30 min	8186	73.9	2982	36.4	4353	53.2	851	10.4	4229	51.7	3957	48.3
< 30 min	2894	26.1	774	26.7	1644	56.8	476	16.4	1365	47.2	1529	52.8

Abbreviations: GDS = Geriatric Depression Scale; JPY = Japanese Yen.

**TABLE 2:** Incident of disability and death in each transition by oral frailty and dental visit status after imputation (*n* = 11 080).

Baseline oral frailty status	Dental visit	Transition 1			Transition 2^a^			Transition 3		
		Healthy to disabled			Healthy to death			Disabled to death		
		Overall	Onset (*n*)	%	Overall	Onset (*n*)	%	Overall	Onset (*n*)	%
		11 080	1170	10.6	11 080	1228	11.1	1170	481	41.1
Non‐OF	Visit	2032	120	5.9	2032	122	6.0	120	44	36.7
	No visit	1724	131	7.6	1724	138	8.0	131	54	41.2
Pre‐OF	Visit	2930	304	10.4	2930	310	10.6	304	123	40.5
	No visit	3067	402	13.1	3067	430	14.0	402	169	42.0
OF	Visit	632	80	12.7	632	84	13.3	80	29	36.3
	No visit	695	133	19.1	695	144	20.7	133	62	46.6

^a^ Transition 2 in this table includes the onset of those who experience disability before death too.

Table 3 shows the results of the multistate survival model. OF status was associated with a higher risk of transitions 1 and 2, but not transition 3 (disability to death). In the fully adjusted model (Model III), pre‐OF individuals had a 1.19 times higher risk of disability (95% CI: 1.03–1.38), and OF individuals had a 1.23 times higher risk (95% CI: 1.01–1.50). For transition to death, pre‐OF individuals had a 1.22 times higher risk (95% CI: 1.02–1.46), and OF individuals had a 1.34 times higher risk (95% CI: 1.05–1.71). Those with no dental visits were associated with a higher risk of transitioning from healthy to disabled (HR: 1.16; 95% CI: 1.03–1.30) and from healthy to death (HR: 1.25; 95% CI: 1.08–1.45).

**TABLE 3:** Multistate survival model: hazard ratio of oral frailty and dental visit with disability or death in each transition, after imputation (*n* = 11 080).

Oral frailty status	Model I^a^		Model II^b^		Model III^c^	
	HR	95% CI	HR	95% CI	HR	95% CI
Transition 1: Healthy to disabled						
Non‐OF	Ref		Ref		Ref	
Pre‐OF	1.35**	1.16–1.56	1.33**	1.15–1.54	1.19**	1.03–1.38
OF	1.59**	1.32–1.91	1.57**	1.31–1.89	1.23**	1.01–1.50
Dental visit			Ref		Ref	
No dental visit			1.20**	1.06–1.34	1.16**	1.03–1.30
Transition 2: Healthy to death						
Non‐OF	Ref		Ref		Ref	
Pre‐OF	1.4**	1.18–1.68	1.39**	1.17–1.66	1.22**	1.02–1.46
OF	1.75**	1.39–2.20	1.73**	1.38–2.18	1.34**	1.05–1.71
Dental visit			Ref		Ref	
No dental visit			1.29**	1.12–1.49	1.25**	1.08–1.45
Transition 3: Disabled to dead						
Non‐OF	Ref		Ref		Ref	
Pre‐OF	1.06	0.84–1.33	1.06	0.84–1.33	1.05	0.83–1.33
OF	1.04	0.78–1.39	1.03	0.78–1.38	1.03	0.77–1.38
Dental visit			Ref		Ref	
No dental visit			1.08	0.90–1.30	1.08	0.89–1.30

*Note:* Estimated using Royston‐Parmar flexible Parametric Model.

Abbreviations: CI = confidence interval; HR = hazard ratio.

** Significant at 0.001.

^a^ Model I: Adjusted with age and sex.

^b^ Mode II: Adjusted with dental visit, age, and sex.

^c^ Model III: Adjusted with dental visit and all covariates (age, sex, marital status, living arrangement, education level, employment status, household income, BMI, depressive symptoms, hypertension, diabetes, stroke, cancer, dementia, smoking status, alcohol consumption, walking time).

**FIGURE 1:**
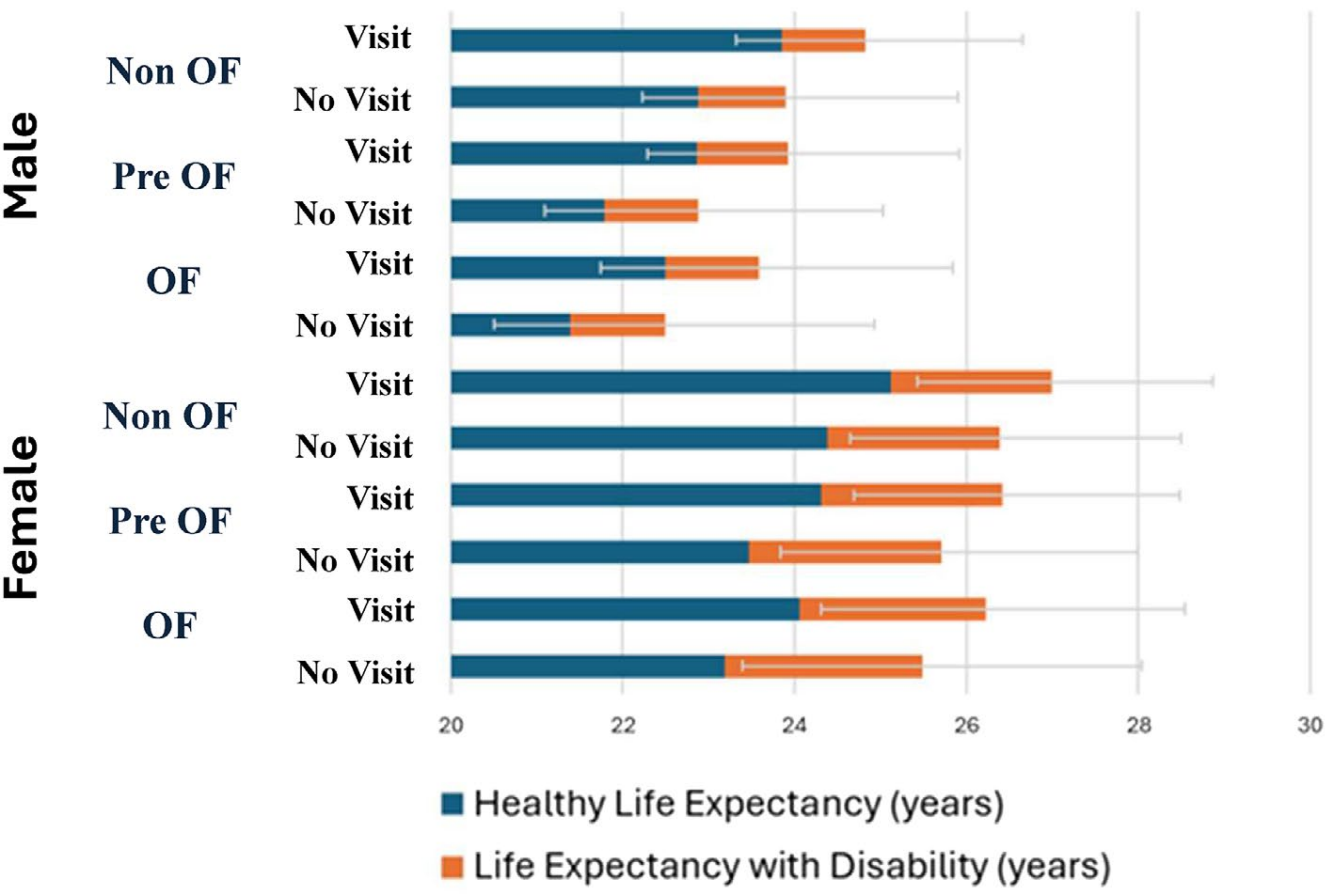
Difference in total life expectancy, healthy life expectancy, and life expectancy with disability at 65 years old with different oral frailty status and dental visit (in years) for each sex, after imputation (*n* = 11 080). Estimated using Royston‐Parmar flexible Parametric Model. Estimated model adjusted with the covariates prevalence (age, sex, marital status, living arrangement, education level, employment status, household income, BMI, depressive symptoms, hypertension, diabetes, stroke, cancer, dementia, smoking status, alcohol consumption, walking time) to show population average estimates. CI = confidence interval; HLE = healthy life expectancy; LED = life expectancy with disability; OF = oral frailty; TLE = total life expectancy.

The fully adjusted model estimated TLE at age 65 as 23.80 years (95% CI: 22.1–25.70; HLE: 22.75, 95% CI: 21.57–24.00) for males and 26.33 years (95% CI: 24.66–28.33; HLE: 24.25, 95% CI: 23.22–24.25) for females (Table S4). Table 4 shows the estimated HLE and TLE at age 65 and their differences between each exposure. Non‐OF males had the highest HLE (23.39 years), followed by pre‐OF (22.35 years) and OF (21.96 years). Non‐OF females had the highest HLE (24.77 years), followed by pre‐OF (23.91 years) and OF (23.64 years). Those who visited a dentist in the past 6 months had approximately 1 year longer HLE compared to those who did not visit a dentist, within the same sex and OF category (Figure 1 and Table S5). The results of our sensitivity analyses in different distributions were relatively consistent with the trend of our main results. However, although all sensitivity analyses showed similar hazard ratios for each transition, the Gompertz distribution and the complete‐case analysis estimated shorter years (Tables S6–S17).

**TABLE 4:** Difference in total life expectancy, healthy life expectancy, and life expectancy with disability at 65 years old with different sex and oral frailty status (in years), after imputation (*n* = 11 080).

Sex	Oral frailty status	Healthy life expectancy (HLE)	HLE 95% CI (lower–higher)	Difference in HLE	Life expectancy with disability (LED)	LED 95% CI (lower–higher)	Differences in LED	Total life expectancy (TLE)	TLE 95% CI (lower–higher)	Difference in TLE
Male	Non‐OF	23.39	22.22–24.61	1.42	1	0.60–1.65	−0.1	24.38	23.87–26.26	1.32
	Pre‐OF	22.35	21.09–23.68	0.38	1.08	0.66–1.77	−0.02	23.42	22.86–25.45	0.36
	OF	21.96	20.53–23.49	Ref	1.1	0.65–1.88	Ref	23.06	22.41–25.37	Ref
Female	Non‐OF	24.77	23.76–25.82	1.02	1.94	1.32–2.84	−0.29	26.71	26.60–28.66	0.83
	Pre‐OF	23.91	22.82–25.05	0.27	2.17	1.49–3.15	−0.06	26.08	25.97–28.21	0.21
	OF	23.64	22.41–24.94	Ref	2.23	1.49–3.33	Ref	25.87	25.74–28.27	Ref

*Note:* Estimated using Royston‐Parmar flexible Parametric Model (Model III). Estimated model adjusted with the covariates prevalence (age, sex, marital status, living arrangement, education level, employment status, household income, BMI, depressive symptoms, hypertension, diabetes, stroke, cancer, dementia, smoking status, alcohol consumption, walking time) to show population average estimates.

Abbreviations: CI = confidence interval; HLE = healthy life expectancy; LED = life expectancy with disability; TLE = total life expectancy.

## 7 Discussion

This study showed that a dose–response association was observed between OF and HLE, suggesting the significant impact of poor oral health and function on the well‐being of older adults. Females generally had longer HLE and TLE, but also more LED. Additionally, those who visited a dentist in the past 6 months had better outcomes for both TLE and HLE, regardless of gender and OF status. Dental visits were associated with approximately 1 year longer HLE.

In 2023, Japan's Ministry of Health, Labor, and Welfare reported average TLE at age 65 of 19.52 years for men and 24.38 years for women [2]. In this study, TLE was 23.80 (HLE: 22.75) years for men and 26.33 (HLE: 24.25) years for women, higher than the national estimates. This difference likely reflects the study's population, which included only independent older adults, excluding institutionalized individuals and those already certified as disabled before follow‐up. The relatively short follow‐up period may also contribute to this variance [11, 19]. Despite higher absolute estimates, the present result is consistent with previous studies that showed that there was an association between oral status with disability [5, 12, 26] and mortality [6, 9, 27], thereby affecting HLE [5, 11, 17]. Another study also showed that older adults who do not use dental services have a higher risk of frailty [15], as well as a reduced HLE and TLE across oral health groups [5].

The association between OF and HLE may be explained by several mechanisms [8]. First, OF, which also includes dysphagia, can lead to malnutrition, worsening frailty, and physical weakness [28, 29, 30]. Safe swallowing depends on coordinated systems, but frailty‐related muscle weakness and inflammation increase swallowing difficulty [17], reducing independence and quality of life. Poor oral health, such as tooth decay and dry mouth, disrupts oral bacteria, raising the risk of odontogenic infections or pneumonia, which can lead to severe complications, disability, and even death [30, 31]. Dental problems like tooth decay, tooth loss, periodontitis, and oral pain contribute to poor oral function and OF, leading to worsening overall health and social isolation, which are also significant risk factors for disability and mortality in older adults [17, 19, 30]. These findings suggest that poor oral health is not only a localized problem, but part of a broader decline in physical and cognitive function that directly affects longevity and well‐being [8, 17, 32].

Given these associations, maintaining oral health goes beyond tooth loss. While OF is a condition that can be improved with the right care, aging and increasing OF often make dental treatment more challenging [14, 33, 34]. For non‐OF older adults, dental care may help prevent oral problems that can lead to serious health conditions and disability. In pre‐OF and OF individuals, it becomes even more important to manage pain, support rehabilitation, and ensure proper nutrition while also slowing further physical and cognitive decline [15]. Regular dental care over time may help reduce the risk of oral infections, detect systemic diseases, and improve overall health [5, 12, 14, 35]. Good oral health, through preventive care [36], regular dental visits [14], functional training [37], and policies supporting lifelong oral function, plays a key role in preventing OF and promoting healthy aging with extended HLE.

This study has several strengths. First, this study was able to account for confounding factors in the prediction of HLE differences according to OF [5, 19]. Multistate modeling, compared with Cox models, handles multiple states and transitions, accounts for competing risks, and provides hazard ratios for multiple events [18, 24]. Additionally, this study utilizes data on disability onset and death sourced from municipal records, which have less attrition bias and are more reliable than self‐reported data [11].

However, this study had several limitations. Exposure variables and covariates were self‐reported, which may be subject to recall and social desirability bias [38]. Key measures such as the number of teeth have been validated with clinical examination [39], but dry mouth and swallowing difficulty have not [8]. Additionally, the use of a single question on speaking difficulty in JAGES may not fully capture other oral motor functions and differs from the validated OF‐5 questionnaire [7, 9, 10]. After trying multiple cut‐offs, including the current binary consensus (Table S18), our main analyses used a three‐category classification instead of a two‐category model [7] to capture more detailed trends in the OF progression. The use of different items and categorization from the OF‐5 consensus may limit comparability with other studies.

OF status and other time‐varying confounders were only assessed at baseline, limiting our ability to monitor changes over time. Some participants may have recovered from OF through dental treatment, likely underestimating the OF–HLE relationship. Although unmeasured confounders may affect the results, our analysis accounted for a rich set of major confounders, with E‐values [40] ranged from 1.75 to 2.23 for OF and dental visits across three transitions. Additionally, a 6‐year follow‐up period may be relatively short for assessing the lifespan of younger older adults, and a longer follow‐up period may be needed [2, 11, 19].

## 8 Conclusion

OF status is significantly associated with lower HLE, emphasizing oral health's importance in older adults' well‐being. Dental visits are associated with longer HLE for both sexes, regardless of OF status. These findings suggest the need for public health policies promoting oral health, which could significantly improve the quality of life and reduce disability burden among older adults.

## Author Contributions

**S.Kh.:** conceptualization, methodology, data curation, formal analysis, writing – original draft. **S.Ki.:** methodology, formal analysis, writing – review and editing, supervision. **Y.M.:** methodology, formal analysis, writing – review and editing, supervision. **M.I.:** writing – review and editing, supervision. **J.A.:** conceptualization, methodology, data curation, formal analysis, supervision, funding acquisition.

## Ethics Statement

The JAGES 2016 study followed ethical guidelines approved by the ethics committees of the National Center for Geriatrics and Gerontology (Approval No. 992), Chiba University (Approval No. 2493), and the Institute of Science Tokyo (D2022‐040‐01). Participants received a written explanation and completed the questionnaire if they agreed to participate.

## Conflicts of Interest

The authors declare no conflicts of interest.

## Supporting information


**Figure S1:** Flowchart of study participants after imputation.
**Figure S2:** Disability‐death model: Trajectory from healthy to disabled and death.
**Figure S3:** Flowchart of study participant for complete case analysis (before imputation).
**Table S1:** Baseline demographic and characteristic based on oral frailty status and dental visit in complete case before imputation (*n* = 5860).
**Table S2:** Baseline demographic and characteristic based on oral frailty status and dental visit with missing values, before excluding individuals who required care in baseline (*n* = 11 687).
**Table S3:** Incident of disability and death in each transition by characteristics after imputation (*n* = 11.080).
**Table S4:** Expected life expectancy at 65 years old (years) for each sex, after imputation (*n* = 11 080).
**Table S5:** Difference in total life expectancy, healthy life expectancy, and life expectancy with disability at 65 years old with different oral frailty status and dental visit (in years) for each sex, after imputation (*n* = 11 080).
**Table S6:** Multistate model: hazard ratio of oral frailty and dental visit with disability or death in each transition excluding a 1‐year onset from baseline, after imputation (*n* = 10 918).
**Table S7:** Difference in total life expectancy, healthy life expectancy, and life expectancy with disability at 65 years old with different oral frailty status (in years) excluding a 1‐year onset from baseline, after imputation (*n* = 10 918).
**Table S8:** Difference in total life expectancy, healthy life expectancy, and life expectancy with disability at 65 years old with different oral frailty status and dental visit (in years) for each sex excluding a 1‐year onset from baseline, after imputation (*n* = 10 918).
**Table S9:** Multistate model: hazard ratio of oral frailty and dental visit with disability or death in each transition using Gompertz Distribution, after imputation (*n* = 11 080).
**Table S10:** Difference in total life expectancy, healthy life expectancy, and life expectancy with disability at 65 years old with different oral frailty status (in years), using Gompertz distribution (*n* = 11 080).
**Table S11:** Difference in total life expectancy, healthy life expectancy, and life expectancy with disability at 65 years old with different oral frailty status and dental visit (in years) for each sex using Gompertz distribution, after imputation (*n* = 11 080).
**Table S12:** Multistate model: hazard ratio of oral frailty and dental visit with disability or death in each transition using Weibull distribution, after imputation (*n* = 11 080).
**Table S13:** Difference in total life expectancy, healthy life expectancy, and life expectancy with disability at 65 years old with different sex and oral frailty status (in years) using Weibull distribution, after imputation (*n* = 11 080).
**Table S14:** Difference in total life expectancy, healthy life expectancy, and life expectancy with disability at 65 years old with different oral frailty status and dental visit (in years) for each sex using Weibull distribution, after imputation (*n* = 11 080).
**Table S15:** Multistate model: Hazard ratio of oral frailty and dental visit with disability or death in each transition, in complete case analysis (*n* = 5890).
**Table S16:** Difference in total life expectancy, healthy life expectancy, and life expectancy with disability at 65 years old with different oral frailty status (years) in complete case analysis (*n* = 5890).
**Table S17:** Difference in total life expectancy, healthy life expectancy, and life expectancy with disability at 65 years old with different oral frailty status and dental visit in complete case analysis (*n* = 5890).
**Table S18:** Multistate model: Hazard ratio of oral frailty and dental visit with disability or death in each transition, comparison of hazard ratios (HR) for different cut‐off points of oral frailty (*n* = 11 080).

## Data Availability

The data that support the findings of this study are available from Japan Gerontological Evaluation Study (JAGES). Restrictions apply to the availability of these data, which were used under license for this study. Data are available from https://www.jages.net/data_application/ with the permission of Japan Gerontological Evaluation Study (JAGES).
