# Supplementary material for: Oral Frailty, Dental Visits, and Healthy Life Expectancy: A 6‐Year Prospective Cohort Among Japanese Older Adults
Source: Geriatr Gerontol Int. 2025 Oct 21;25(12):1884–93. doi: 10.1111/ggi.70230 (PMC12719132; doi:10.1111/ggi.70230)
Supplement: Supplementary file 1 — Figure S1: Flowchart of study participants after imputation. Figure S2: Disability‐death model: Trajectory from healthy to disabled and death. Figure S3: Flowchart of study participant for complete case analysis (before imputation). Table S1: Baseline demographic and characteristic based on oral frailty status and dental visit in complete case before imputation (n = 5860). Table S2: Baseline demographic and characteristic based on oral frailty status and dental visit with missing values, before excluding individuals who required care in baseline (n = 11 687). Table S3: Incident of disability and death in each transition by characteristics after imputation (n = 11.080). Table S4: Expected life expectancy at 65 years old (years) for each sex, after imputation (n = 11 080). Table S5: Difference in total life expectancy, healthy life expectancy, and life expectancy with disability at 65 years old with different oral frailty status and dental visit (in years) for each sex, after imputation (n = 11 080). Table S6: Multistate model: hazard ratio of oral frailty and dental visit with disability or death in each transition excluding a 1‐year onset from baseline, after imputation (n = 10 918). Table S7: Difference in total life expectancy, healthy life expectancy, and life expectancy with disability at 65 years old with different oral frailty status (in years) excluding a 1‐year onset from baseline, after imputation (n = 10 918). Table S8: Difference in total life expectancy, healthy life expectancy, and life expectancy with disability at 65 years old with different oral frailty status and dental visit (in years) for each sex excluding a 1‐year onset from baseline, after imputation (n = 10 918). Table S9: Multistate model: hazard ratio of oral frailty and dental visit with disability or death in each transition using Gompertz Distribution, after imputation (n = 11 080). Table S10: Difference in total life expectancy, healthy life expectancy, and life expectancy with disabilit [file GGI-25-1884-s001.docx]

Supplementary Materials

Title: **Oral Frailty, Dental Visits, and Healthy Life Expectancy: a 6 years prospective cohort among Japanese Older Adults**

**Supplementary Figure 1**. Flowchart of study participants after imputation.

**Supplementary Figure 2**. Disability-Death model: Trajectory from Healthy to Disabled and Death

**Supplementary Figure 3.** Flowchart of study participant for complete case analysis (before imputation)

**Supplementary Table 1**. Baseline Demographic and Characteristic Based on Oral Frailty Status and Dental Visit in complete case before imputation (n=5,860)

**Supplementary Table 2.** Baseline Demographic and Characteristic Based on Oral Frailty Status and Dental Visit with Missing Values, before Excluding Individuals Who Required Care in Baseline (n=11,687)

**Supplementary Table 3.** Incident of Disability and Death in each Transition by Characteristics After Imputation (n=11.080)

**Supplementary Table 4.** Expected Life Expectancy at 65 years old (years) for Each Sex, after imputation (n=11,080)

**Supplementary Table 5.** Difference in Total Life Expectancy, Healthy Life Expectancy, and Life Expectancy with Disability at 65 years old with different Oral Frailty Status and Dental Visit (in years) for Each Sex, after imputation (n=11,080)

**Supplementary Table 6.** Multistate Model: Hazard Ratio of Oral Frailty and Dental Visit with Disability or Death in Each Transition excluding a 1-year onset from baseline, after imputation (n=10,918)

**Supplementary Table 7.** Difference in Total Life Expectancy, Healthy Life Expectancy, and Life Expectancy with Disability at 65 years old with different Oral Frailty Status (in years) excluding a 1-year onset from baseline, after imputation (n=10,918)

**Supplementary Table 8.** Difference in Total Life Expectancy, Healthy Life Expectancy, and Life Expectancy with Disability at 65 years old with different Oral Frailty Status and Dental Visit (in years) for Each Sex excluding a 1-year onset from baseline, after imputation (n=10,918)

**Supplementary Table 9.** Multistate Model: Hazard Ratio of Oral Frailty and Dental Visit with Disability or Death in Each Transition using Gompertz Distribution, after imputation (n=11,080)

**Supplementary Table 10.** Difference in Total Life Expectancy, Healthy Life Expectancy, and Life Expectancy with Disability at 65 years old with different Oral Frailty Status (in years), using Gompertz distribution (n=11,080)

**Supplementary Table 11.** Difference in Total Life Expectancy, Healthy Life Expectancy, and Life Expectancy with Disability at 65 years old with different Oral Frailty Status and Dental Visit (in years) for Each Sex using Gompertz distribution, after imputation (n=11,080)

**Supplementary Table 12.** Multistate Model: Hazard Ratio of Oral Frailty and Dental Visit with Disability or Death in Each Transition using Weibull Distribution, after imputation (n=11,080)

**Supplementary Table 13.** Difference in Total Life Expectancy, Healthy Life Expectancy, and Life Expectancy with Disability at 65 years old with different Sex and Oral Frailty Status (in years) using Weibull Distribution, after imputation (n=11,080)

**Supplementary Table 14**. Difference in Total Life Expectancy, Healthy Life Expectancy, and Life Expectancy with Disability at 65 years old with different Oral Frailty Status and Dental Visit (in years) for Each Sex using Weibull distribution, after imputation (n=11,080)

**Supplementary Table 15.** Multistate Model: Hazard Ratio of Oral Frailty and Dental Visit with Disability or Death in Each Transition, in Complete Case Analysis (n=5,890)

**Supplementary Table 16.** Difference in Total Life Expectancy, Healthy Life Expectancy, and Life Expectancy with Disability at 65 years old with different Oral Frailty Status (years) in Complete Case Analysis (n=5,890)

**Supplementary Table 17.** Difference in Total Life Expectancy, Healthy Life Expectancy, and Life Expectancy with Disability at 65 years old with different Oral Frailty Status and Dental Visit in Complete Case Analysis (n=5,890*)*

**Supplementary Table 18.** Multistate Model: Hazard Ratio of Oral Frailty and Dental Visit with Disability or Death in Each Transition, Comparison of Hazard Ratios (HR) for Different Cut-off Points of Oral Frailty (n=11,080*)*


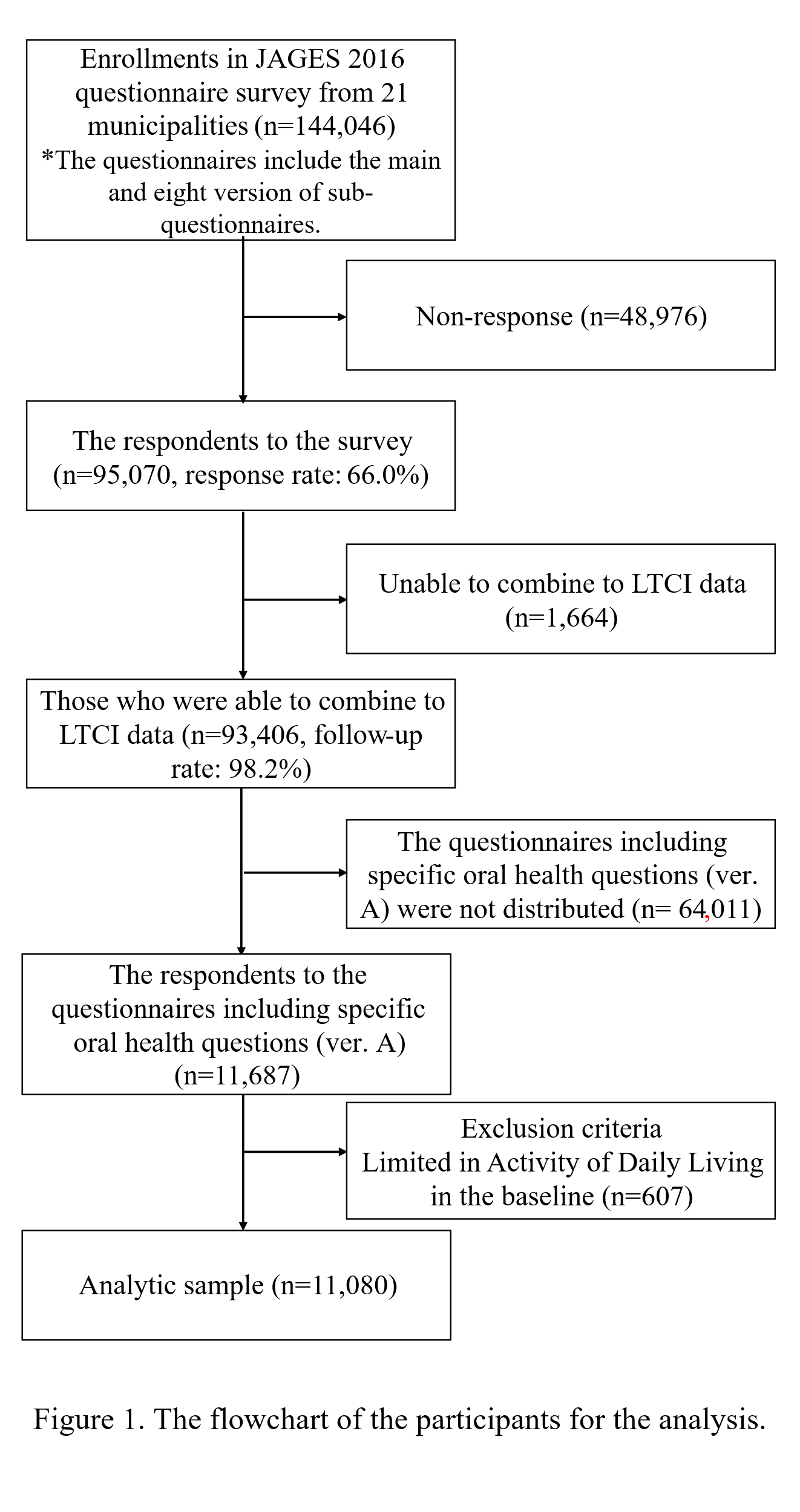


*LTCI: Long-term Care Insurance*

**Supplementary Figure 1**. Flowchart of study participant after imputation.


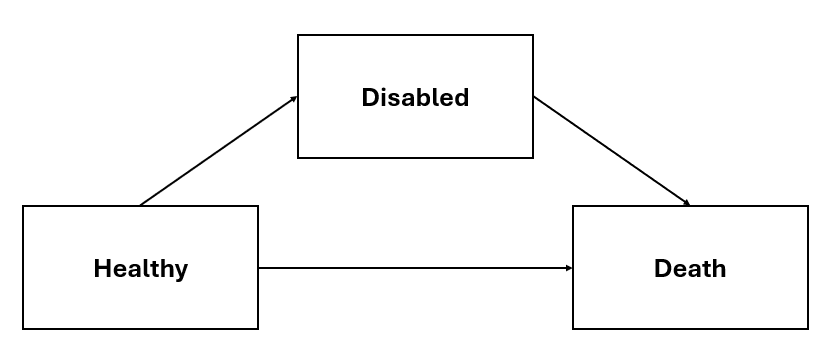


**Supplementary Figure 2**. Disability-Death model: Trajectory from Healthy to Disabled and Death


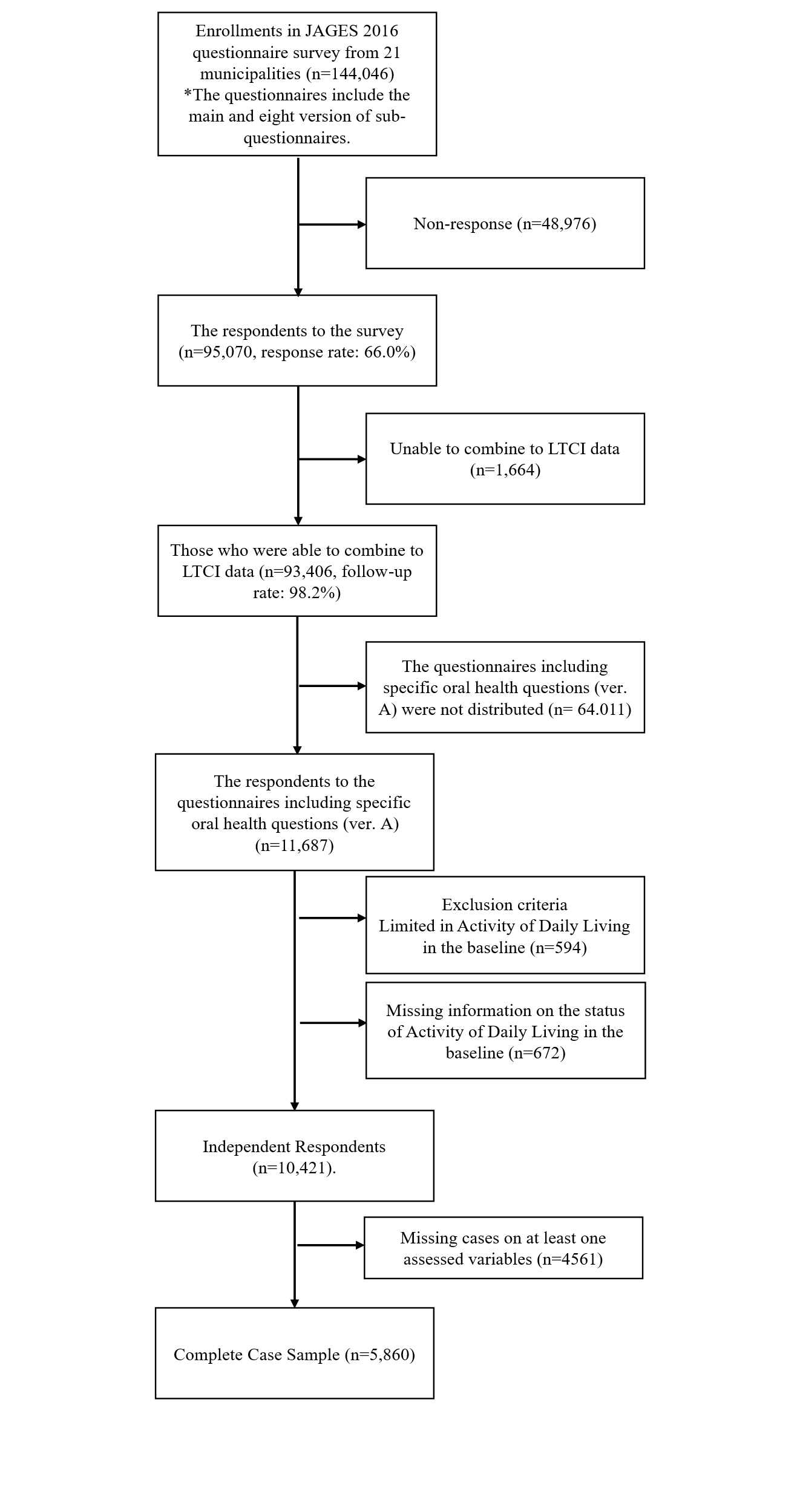


*LTCI: Long-term Care Insurance*

**Supplementary Figure 3.** Flowchart of study participant for complete case analysis (before imputation)

| **Supplementary Table 1**. Baseline Demographic and Characteristic Based on Oral Frailty Status and Dental Visit in complete case before imputation (n=5,860) | | | | | | | | | | | | | |  |
| --- | --- | --- | --- | --- | --- | --- | --- | --- | --- | --- | --- | --- | --- | --- |
|  | **Overall**  **5,860** | | **Oral Frailty Status** | | | | | | | **Dental Visit in the last 6 months** | | | | |
|  |  |  | Non OF(0) | | Pre OF (1-2) | | | OF (3-5) | | No Visit | | Visit | | |
|  | n | %col | n | %row | n | %row | n | | %row | n | %row | n | %row | |
|  |  |  | 2211 | 37.7 | 3000 | 51.2 | 649 | | 11.1 | 2,681 | 45.8 | 3,179 | 54.2 | |
| **Age** |  |  |  |  |  |  |  | |  |  |  |  |  | |
| 65-69 | 2002 | 34.2 | 932 | 46.6 | 899 | 44.9 | 171 | | 8.5 | 981 | 49.0 | 1021 | 51.0 | |
| 70-74 | 1690 | 28.8 | 646 | 38.2 | 882 | 52.2 | 162 | | 9.6 | 710 | 42.0 | 980 | 58.0 | |
| 75-79 | 1271 | 21.7 | 420 | 33.0 | 683 | 53.7 | 168 | | 13.2 | 535 | 42.1 | 736 | 57.9 | |
| 80-84 | 643 | 11.0 | 174 | 27.1 | 365 | 56.8 | 104 | | 16.2 | 310 | 48.2 | 333 | 51.8 | |
| 85+ | 254 | 4.3 | 39 | 15.4 | 171 | 67.3 | 44 | | 17.3 | 145 | 57.1 | 109 | 42.9 | |
| **Sex** |  |  |  |  |  |  |  | |  |  |  |  |  | |
| Female | 2642 | 45.1 | 1042 | 39.4 | 1339 | 50.7 | 261 | | 9.9 | 1118 | 42.3 | 1524 | 57.7 | |
| Male | 3218 | 54.9 | 1169 | 36.3 | 1661 | 51.6 | 388 | | 12.1 | 1563 | 48.6 | 1655 | 51.4 | |
| **Marital Status** |  |  |  |  |  |  |  | |  |  |  |  |  | |
| Married | 4562 | 77.8 | 1811 | 39.7 | 2275 | 49.9 | 476 | | 10.4 | 2022 | 44.3 | 2540 | 55.7 | |
| Not Married | 1298 | 22.2 | 400 | 30.8 | 725 | 55.9 | 173 | | 13.3 | 659 | 50.8 | 639 | 49.2 | |
| **Living Arrangement** |  |  |  |  |  |  |  | |  |  |  |  |  | |
| Living Alone | 777 | 13.3 | 251 | 32.3 | 430 | 55.3 | 96 | | 12.4 | 384 | 49.4 | 393 | 50.6 | |
| not Living Alone | 5083 | 86.7 | 1960 | 38.6 | 2570 | 50.6 | 553 | | 10.9 | 2297 | 45.2 | 2786 | 54.8 | |
| **Educational Attainment** |  |  |  |  |  |  |  | |  |  |  |  |  | |
| <=9 years | 1503 | 25.6 | 434 | 28.9 | 833 | 55.4 | 236 | | 15.7 | 793 | 52.8 | 710 | 47.2 | |
| >9 years | 4357 | 74.4 | 1777 | 40.8 | 2167 | 49.7 | 413 | | 9.5 | 1888 | 43.3 | 2469 | 56.7 | |
| **Employment** |  |  |  |  |  |  |  | |  |  |  |  |  | |
| Employed | 1745 | 29.8 | 693 | 39.7 | 875 | 50.1 | 177 | | 10.1 | 857 | 49.1 | 888 | 50.9 | |
| Not Employed | 4115 | 70.2 | 1518 | 36.9 | 2125 | 51.6 | 472 | | 11.5 | 1824 | 44.3 | 2291 | 55.7 | |
| **Equivalent Income** |  |  |  |  |  |  |  | |  |  |  |  |  | |
| <2 millions/year | 1191 | 20.3 | 332 | 27.9 | 643 | 54.0 | 216 | | 18.1 | 638 | 53.6 | 553 | 46.4 | |
| 2-4 millions/year | 2580 | 44.0 | 999 | 38.7 | 1338 | 51.9 | 243 | | 9.4 | 1159 | 44.9 | 1421 | 55.1 | |
| >4 millions/year | 2089 | 35.6 | 880 | 42.1 | 1019 | 48.8 | 190 | | 9.1 | 884 | 42.3 | 1205 | 57.7 | |
| **BMI** |  |  |  |  |  |  |  | |  |  |  |  |  | |
| Underweight | 368 | 6.3 | 113 | 30.7 | 200 | 54.3 | 55 | | 14.9 | 179 | 48.6 | 189 | 51.4 | |
| Normal weight | 4104 | 70.0 | 1596 | 38.9 | 2070 | 50.4 | 438 | | 10.7 | 1823 | 44.4 | 2281 | 55.6 | |
| Overweight | 1388 | 23.7 | 502 | 36.2 | 730 | 52.6 | 156 | | 11.2 | 679 | 48.9 | 709 | 51.1 | |
| **Depressive Symptoms** |  |  |  |  |  |  |  | |  |  |  |  |  | |
| No Depression | 4713 | 80.4 | 1921 | 40.8 | 2386 | 50.6 | 406 | | 8.6 | 2111 | 44.8 | 2602 | 55.2 | |
| Tendency toward Depression | 951 | 16.2 | 254 | 26.7 | 511 | 53.7 | 186 | | 19.6 | 465 | 48.9 | 486 | 51.1 | |
| Depression | 196 | 3.3 | 36 | 18.4 | 103 | 52.6 | 57 | | 29.1 | 105 | 53.6 | 91 | 46.4 | |
| **Dementia** |  |  |  |  |  |  |  | |  |  |  |  |  | |
| No | 5843 | 99.7 | 2205 | 37.7 | 2995 | 51.3 | 643 | | 11.0 | 2675 | 45.8 | 3168 | 54.2 | |
| Yes | 17 | 0.3 | 6 | 35.3 | 5 | 29.4 | 6 | | 35.3 | 6 | 35.3 | 11 | 64.7 | |
| **Hypertension** |  |  |  |  |  |  |  | |  |  |  |  |  | |
| No | 3287 | 56.1 | 1295 | 39.4 | 1649 | 50.2 | 343 | | 10.4 | 1488 | 45.3 | 1799 | 54.7 | |
| Yes | 2573 | 43.9 | 916 | 35.6 | 1351 | 52.5 | 306 | | 11.9 | 1193 | 46.4 | 1380 | 53.6 | |
| **Diabetes** |  |  |  |  |  |  |  | |  |  |  |  |  | |
| No | 5098 | 87.0 | 1968 | 38.6 | 2570 | 50.4 | 560 | | 11.0 | 2315 | 45.4 | 2783 | 54.6 | |
| Yes | 762 | 13.0 | 243 | 31.9 | 430 | 56.4 | 89 | | 11.7 | 366 | 48.0 | 396 | 52.0 | |
| **Stroke** |  |  |  |  |  |  |  | |  |  |  |  |  | |
| No | 5711 | 97.5 | 2162 | 37.9 | 2921 | 51.1 | 628 | | 11.0 | 2618 | 45.8 | 3093 | 54.2 | |
| Yes | 149 | 2.5 | 49 | 32.9 | 79 | 53.0 | 21 | | 14.1 | 63 | 42.3 | 86 | 57.7 | |
| **Cancer** |  |  |  |  |  |  |  | |  |  |  |  |  | |
| No | 5611 | 95.8 | 2141 | 38.2 | 2854 | 50.9 | 616 | | 11.0 | 2577 | 45.9 | 3034 | 54.1 | |
| Yes | 249 | 4.2 | 70 | 28.1 | 146 | 58.6 | 33 | | 13.3 | 104 | 41.8 | 145 | 58.2 | |
| **Smoking** |  |  |  |  |  |  |  | |  |  |  |  |  | |
| Non smoker | 3200 | 54.6 | 1356 | 42.4 | 1555 | 48.6 | 289 | | 9.0 | 1362 | 42.6 | 1838 | 57.4 | |
| Past Smoker | 1977 | 33.7 | 680 | 34.4 | 1051 | 53.2 | 246 | | 12.4 | 914 | 46.2 | 1063 | 53.8 | |
| Current Smoker | 683 | 11.7 | 175 | 25.6 | 394 | 57.7 | 114 | | 16.7 | 405 | 59.3 | 278 | 40.7 | |
| **Drinking** |  |  |  |  |  |  |  | |  |  |  |  |  | |
| Non Drinker | 2668 | 45.5 | 992 | 37.2 | 1393 | 52.2 | 283 | | 10.6 | 1184 | 44.4 | 1484 | 55.6 | |
| Past Drinker | 622 | 10.6 | 181 | 29.1 | 354 | 56.9 | 87 | | 14.0 | 334 | 53.7 | 288 | 46.3 | |
| Current Drinker | 2570 | 43.9 | 1038 | 40.4 | 1253 | 48.8 | 279 | | 10.9 | 1163 | 45.3 | 1407 | 54.7 | |
| **Walking Time** |  |  |  |  |  |  |  | |  |  |  |  |  | |
| >= 30 mins | 4434 | 75.7 | 1790 | 40.4 | 2221 | 50.1 | 423 | | 9.5 | 1959 | 44.2 | 2475 | 55.8 | |
| <30 mins | 1426 | 24.3 | 421 | 29.5 | 779 | 54.6 | 226 | | 15.8 | 722 | 50.6 | 704 | 49.4 | |

**Supplementary Table 2.** Baseline Demographic and Characteristic Based on Oral Frailty Status and Dental Visit with Missing Values, before Excluding Individuals Who Had Limited Activity of Daily Living (n=11,687)

|  | **Overall** | **11,687** | **Oral Frailty Status** | | | | | | | | | | **Dental Visit in the last 6 months** | | | |
| --- | --- | --- | --- | --- | --- | --- | --- | --- | --- | --- | --- | --- | --- | --- | --- | --- |
|  |  |  | No Frailty (0) | | | Prefrail (1-2) | | | Frailty (3-5) | | *missing* | | No Visit | | Visit | |
|  | n | %col | n | %row | n | | %row | n | | %row | n | %row | n | %row | n | %row |
|  |  |  | 3,820 | 32.7 | 1,273 | | 10.9 | 602 | | 5.2 |  |  | 5,832 | 49.9 | 5,855 | 50.1 |
| **Limited ADL** |  |  |  |  |  | |  |  | |  |  |  |  |  |  |  |
| Yes | 594 | 5.1 | 59 | 9.9 | 295 | | 49.7 | 181 | | 30.5 | 59 | 9.9 | 236 | 39.7 | 358 | 60.3 |
| No | 10,421 | 89.2 | 3,617 | 34.7 | 5,399 | | 51.8 | 1,015 | | 9.7 | 390 | 3.7 | 5,314 | 51.0 | 5,107 | 49.0 |
| *missing* | 672 | 5.7 | 144 | 21.4 | 298 | | 44.3 | 77 | | 11.5 | 153 | 22.8 | 282 | 42.0 | 390 | 58.0 |
| **Age** |  |  |  |  |  | |  |  | |  |  |  |  |  |  |  |
| 65-69 | 3,289 | 28.1 | 1,428 | 43.4 | 1,532 | | 46.6 | 233 | | 7.1 | 96 | 2.9 | 1,597 | 48.6 | 1,692 | 51.4 |
| 70-74 | 3,173 | 27.1 | 1,095 | 34.5 | 1,677 | | 52.9 | 276 | | 8.7 | 125 | 3.9 | 1,715 | 54.0 | 1,458 | 46.0 |
| 75-79 | 2,741 | 23.5 | 849 | 31.0 | 1,412 | | 51.5 | 335 | | 12.2 | 145 | 5.3 | 1,461 | 53.3 | 1,280 | 46.7 |
| 80-84 | 1,655 | 14.2 | 343 | 20.7 | 918 | | 55.5 | 253 | | 15.3 | 141 | 8.5 | 767 | 46.3 | 888 | 53.7 |
| 85+ | 829 | 7.1 | 105 | 12.7 | 453 | | 54.6 | 176 | | 21.2 | 95 | 11.5 | 292 | 35.2 | 537 | 64.8 |
| *missing* | 0 | 0.0 | 0 | 0 | 0 | | 0 | 0 | | 0 | 0 | 0 | 0 | 0 | 0 | 0 |
| **Sex** |  |  |  |  |  | |  |  | |  |  |  |  |  |  |  |
| Female | 6,218 | 53.2 | 2,066 | 33.2 | 3,130 | | 50.3 | 632 | | 10.2 | 390 | 6.3 | 3,232 | 52.0 | 2,986 | 48.0 |
| Male | 5,469 | 46.8 | 1,754 | 32.1 | 2,862 | | 52.3 | 641 | | 11.7 | 212 | 3.9 | 2,600 | 47.5 | 2,869 | 52.5 |
| missing | 0 | 0.0 | 0 | 0 | 0 | | 0 | 0 | | 0 | 0 | 0 | 0 | 0 | 0 | 0 |
| **Marital Status** |  |  |  |  |  | |  |  | |  |  |  |  |  |  |  |
| Married | 8,424 | 72.1 | 3,017 | 35.8 | 4,235 | | 50.3 | 828 | | 9.8 | 344 | 4.1 | 4,337 | 51.5 | 4,087 | 48.5 |
| Not Married | 3,107 | 26.6 | 774 | 24.9 | 1,679 | | 54.0 | 428 | | 13.8 | 226 | 7.3 | 1,436 | 46.2 | 1,671 | 53.8 |
| *missing* | 156 | 1.3 | 29 | 18.6 | 78 | | 50.0 | 17 | | 10.9 | 32 | 20.5 | 59 | 37.8 | 97 | 62.2 |
| **Living Arrangement** |  |  |  |  |  | |  |  | |  |  |  |  |  |  |  |
| Living Alone | 1,670 | 14.3 | 442 | 26.5 | 910 | | 54.5 | 208 | | 12.5 | 110 | 6.6 | 810 | 48.5 | 860 | 51.5 |
| not Living Alone | 9,293 | 79.5 | 3,197 | 34.4 | 4,710 | | 50.7 | 970 | | 10.4 | 416 | 4.5 | 4,714 | 50.7 | 4,579 | 49.3 |
| *missing* | 724 | 6.2 | 181 | 25.0 | 372 | | 51.4 | 95 | | 13.1 | 76 | 10.5 | 308 | 42.5 | 416 | 57.5 |
| **Educational Attainment** |  |  |  |  |  | |  |  | |  |  |  |  |  |  |  |
| <=9 years | 3,915 | 33.5 | 947 | 24.2 | 2,093 | | 53.5 | 573 | | 14.6 | 302 | 7.7 | 1,685 | 43.0 | 2,230 | 57.0 |
| >9 years | 7,662 | 65.6 | 2,846 | 37.1 | 3,848 | | 50.2 | 688 | | 9.0 | 280 | 3.7 | 4,106 | 53.6 | 3,556 | 46.4 |
| *missing* | 110 | 0.9 | 27 | 24.5 | 51 | | 46.4 | 12 | | 10.9 | 20 | 18.2 | 41 | 37.3 | 69 | 62.7 |
| **Employment** |  |  |  |  |  | |  |  | |  |  |  |  |  |  |  |
| Employed | 2,808 | 24.0 | 1,032 | 36.8 | 1,423 | | 50.7 | 254 | | 9.0 | 99 | 3.5 | 1,397 | 49.8 | 1,411 | 50.2 |
| Not Employed | 6,970 | 59.6 | 2,319 | 33.3 | 3,606 | | 51.7 | 758 | | 10.9 | 287 | 4.1 | 3,703 | 53.1 | 3,267 | 46.9 |
| *missing* | 1,909 | 16.3 | 469 | 24.6 | 963 | | 50.4 | 261 | | 13.7 | 216 | 11.3 | 732 | 38.3 | 1,177 | 61.7 |
| **Equivalent Income** |  |  |  |  |  | |  |  | |  |  |  |  |  |  |  |
| <2 millions/year | 2,621 | 22.4 | 635 | 24.2 | 1,376 | | 52.5 | 416 | | 15.9 | 194 | 7.4 | 1,129 | 43.1 | 1,492 | 56.9 |
| 2-4 millions/year | 4,328 | 37.0 | 1,533 | 35.4 | 2,232 | | 51.6 | 408 | | 9.4 | 155 | 3.6 | 2,280 | 52.7 | 2,048 | 47.3 |
| >4 millions/year | 3,260 | 27.9 | 1,269 | 38.9 | 1,616 | | 49.6 | 275 | | 8.4 | 100 | 3.1 | 1,794 | 55.0 | 1,466 | 45.0 |
| *missing* | 1,478 | 12.6 | 383 | 25.9 | 768 | | 52.0 | 174 | | 11.8 | 153 | 10.4 | 629 | 42.6 | 849 | 57.4 |
| **BMI** |  |  |  |  |  | |  |  | |  |  |  |  |  |  |  |
| Underweight | 876 | 7.5 | 233 | 26.6 | 459 | | 52.4 | 132 | | 15.1 | 52 | 5.9 | 436 | 49.8 | 440 | 50.2 |
| Normal weight | 7,914 | 67.7 | 2,696 | 34.1 | 4,036 | | 51.0 | 828 | | 10.5 | 354 | 4.5 | 4,065 | 51.4 | 3,849 | 48.6 |
| Overweight | 2,897 | 24.8 | 891 | 30.8 | 1,497 | | 51.7 | 313 | | 10.8 | 196 | 6.8 | 1,331 | 45.9 | 1,566 | 54.1 |
| *missing* | 0 | 0.0 | 0 | 0 | 0 | | 0 | 0 | | 0 | 0 | 0 | 0 | 0 | 0 | 0 |
| **Depressive Symptoms** |  |  |  |  |  | |  |  | |  |  |  |  |  |  |  |
| No Depression | 7,517 | 64.3 | 2,848 | 37.9 | 3,855 | | 51.3 | 565 | | 7.5 | 249 | 3.3 | 3,927 | 52.2 | 3,590 | 47.8 |
| Tendency toward Depression | 1,720 | 14.7 | 388 | 22.6 | 917 | | 53.3 | 329 | | 19.1 | 86 | 5.0 | 802 | 46.6 | 918 | 53.4 |
| Depression | 459 | 3.9 | 59 | 12.9 | 235 | | 51.2 | 139 | | 30.3 | 26 | 5.7 | 194 | 42.3 | 265 | 57.7 |
| *missing* | 1,991 | 17.0 | 525 | 26.4 | 985 | | 49.5 | 240 | | 12.1 | 241 | 12.1 | 909 | 45.7 | 1,082 | 54.3 |
| **Dementia** |  |  |  |  |  | |  |  | |  |  |  |  |  |  |  |
| No | 11,166 | 95.5 | 3,687 | 33.0 | 5,774 | | 51.7 | 1,220 | | 10.9 | 485 | 4.3 | 5,613 | 50.3 | 5,553 | 49.7 |
| Yes | 51 | 0.4 | 9 | 17.6 | 23 | | 45.1 | 14 | | 27.5 | 5 | 9.8 | 22 | 43.1 | 29 | 56.9 |
| *missing* | 470 | 4.0 | 124 | 26.4 | 195 | | 41.5 | 39 | | 8.3 | 112 | 23.8 | 197 | 41.9 | 273 | 58.1 |
| **Hypertension** |  |  |  |  |  | |  |  | |  |  |  |  |  |  |  |
| No | 6,141 | 52.5 | 2,132 | 34.7 | 3,076 | | 50.1 | 662 | | 10.8 | 271 | 4.4 | 3,132 | 51.0 | 3,009 | 49.0 |
| Yes | 5,076 | 43.4 | 1,564 | 30.8 | 2,721 | | 53.6 | 572 | | 11.3 | 219 | 4.3 | 2,503 | 49.3 | 2,573 | 50.7 |
| *missing* | 470 | 4.0 | 124 | 26.4 | 195 | | 41.5 | 39 | | 8.3 | 112 | 23.8 | 197 | 41.9 | 273 | 58.1 |
| **Diabetes** |  |  |  |  |  | |  |  | |  |  |  |  |  |  |  |
| No | 9,739 | 83.3 | 3,283 | 33.7 | 4,968 | | 51.0 | 1,060 | | 10.9 | 428 | 4.4 | 4,919 | 50.5 | 4,820 | 49.5 |
| Yes | 1,478 | 12.6 | 413 | 27.9 | 829 | | 56.1 | 174 | | 11.8 | 62 | 4.2 | 716 | 48.4 | 762 | 51.6 |
| *missing* | 470 | 4.0 | 124 | 26.4 | 195 | | 41.5 | 39 | | 8.3 | 112 | 23.8 | 197 | 41.9 | 273 | 58.1 |
| **Stroke** |  |  |  |  |  | |  |  | |  |  |  |  |  |  |  |
| No | 10,891 | 93.2 | 3,606 | 33.1 | 5,624 | | 51.6 | 1,183 | | 10.9 | 478 | 4.4 | 5,473 | 50.3 | 5,418 | 49.7 |
| Yes | 326 | 2.8 | 90 | 27.6 | 173 | | 53.1 | 51 | | 15.6 | 12 | 3.7 | 162 | 49.7 | 164 | 50.3 |
| *missing* | 470 | 4.0 | 124 | 26.4 | 195 | | 41.5 | 39 | | 8.3 | 112 | 23.8 | 197 | 41.9 | 273 | 58.1 |
| **Cancer** |  |  |  |  |  | |  |  | |  |  |  |  |  |  |  |
| No | 10,753 | 92.0 | 3,576 | 33.3 | 5,538 | | 51.5 | 1,165 | | 10.8 | 474 | 4.4 | 5,389 | 50.1 | 5,364 | 49.9 |
| Yes | 464 | 4.0 | 120 | 25.9 | 259 | | 55.8 | 69 | | 14.9 | 16 | 3.4 | 246 | 53.0 | 218 | 47.0 |
| *missing* | 470 | 4.0 | 124 | 26.4 | 195 | | 41.5 | 39 | | 8.3 | 112 | 23.8 | 197 | 41.9 | 273 | 58.1 |
| **Smoking** |  |  |  |  |  | |  |  | |  |  |  |  |  |  |  |
| Non smoker | 6,889 | 58.9 | 2,474 | 35.9 | 3,387 | | 49.2 | 671 | | 9.7 | 357 | 5.2 | 3,621 | 52.6 | 3,268 | 47.4 |
| Past Smoker | 3,375 | 28.9 | 1,036 | 30.7 | 1,839 | | 54.5 | 404 | | 12.0 | 96 | 2.8 | 1,650 | 48.9 | 1,725 | 51.1 |
| Current Smoker | 1,211 | 10.4 | 271 | 22.4 | 697 | | 57.6 | 181 | | 14.9 | 62 | 5.1 | 461 | 38.1 | 750 | 61.9 |
| *missing* | 212 | 1.8 | 39 | 18.4 | 69 | | 32.5 | 17 | | 8.0 | 87 | 41.0 | 100 | 47.2 | 112 | 52.8 |
| **Drinking** |  |  |  |  |  | |  |  | |  |  |  |  |  |  |  |
| Non Drinker | 5,763 | 49.3 | 1,845 | 32.0 | 2,981 | | 51.7 | 624 | | 10.8 | 313 | 5.4 | 2,919 | 50.7 | 2,844 | 49.3 |
| Past Drinker | 1,273 | 10.9 | 309 | 24.3 | 714 | | 56.1 | 194 | | 15.2 | 56 | 4.4 | 556 | 43.7 | 717 | 56.3 |
| Current Drinker | 4,317 | 36.9 | 1,585 | 36.7 | 2,176 | | 50.4 | 426 | | 9.9 | 130 | 3.0 | 2,191 | 50.8 | 2,126 | 49.2 |
| *missing* | 334 | 2.9 | 81 | 24.3 | 121 | | 36.2 | 29 | | 8.7 | 103 | 30.8 | 166 | 49.7 | 168 | 50.3 |
| **Walking Time** |  |  |  |  |  | |  |  | |  |  |  |  |  |  |  |
| >= 30 mins | 8,252 | 70.6 | 2,968 | 36.0 | 4,217 | | 51.1 | 760 | | 9.2 | 307 | 3.7 | 4,265 | 51.7 | 3,987 | 48.3 |
| <30 mins | 3,131 | 26.8 | 794 | 25.4 | 1,664 | | 53.1 | 485 | | 15.5 | 188 | 6.0 | 1,454 | 46.4 | 1,677 | 53.6 |
| *missing* | 304 | 2.6 | 58 | 19.1 | 111 | | 36.5 | 28 | | 9.2 | 107 | 35.2 | 113 | 37.2 | 191 | 62.8 |

ADL = Activity of Daily Living

**Supplementary Table 3.** Incident of Disability and Death in each Transition by Characteristics After Imputation (n=11.080)

|  | **Transition 1** | | | **Transition 2 ^a^** | | | **Transition 3** | | |
| --- | --- | --- | --- | --- | --- | --- | --- | --- | --- |
|  | **Healthy to Disabled** | | | **Healthy to Death** | | | **Disabled to Death** | | |
|  | Overall | Onset (n) | % | Overall | Onset (n) | % | Overall | Onset (n) | % |
|  | 11,080 | 1170 | 10.6 | 11,080 | 1228 | 11.1 | 1,170 | 481 | 41.1 |
| **Oral Frailty** |  |  |  |  |  |  |  |  |  |
| Non OF | 3,756 | 251 | 6.7 | 3,756 | 270 | 7.2 | 251 | 98 | 39.0 |
| Pre OF | 5,997 | 706 | 11.8 | 5,997 | 740 | 12.3 | 706 | 292 | 41.4 |
| Pre OF | 1,327 | 213 | 16.1 | 1,327 | 228 | 17.2 | 213 | 91 | 42.7 |
| **Dental Visit** |  |  |  |  |  |  |  |  |  |
| Yes | 5,594 | 504 | 9.0 | 5,594 | 516 | 9.2 | 504 | 196 | 38.9 |
| No | 5,486 | 666 | 12.1 | 5,486 | 722 | 13.2 | 666 | 285 | 42.8 |
| **Age** |  |  |  |  |  |  |  |  |  |
| 65-69 | 3,218 | 94 | 2.9 | 3,218 | 139 | 4.3 | 94 | 34 | 36.2 |
| 70-74 | 3,082 | 191 | 6.2 | 3,082 | 232 | 7.5 | 191 | 73 | 38.2 |
| 75-79 | 2,586 | 281 | 10.9 | 2,586 | 305 | 11.8 | 281 | 110 | 39.1 |
| 80-84 | 1,505 | 334 | 22.2 | 1,505 | 285 | 18.9 | 334 | 121 | 36.2 |
| 85+ | 689 | 270 | 39.2 | 689 | 277 | 40.2 | 270 | 143 | 53.0 |
| **Sex** |  |  |  |  |  |  |  |  |  |
| Female | 5,858 | 605 | 10.3 | 5,858 | 429 | 7.3 | 605 | 181 | 29.9 |
| Male | 5,222 | 565 | 10.8 | 5,222 | 809 | 15.5 | 565 | 300 | 53.1 |
| **Marrital Status** |  |  |  |  |  |  |  |  |  |
| Married | 8,176 | 736 | 9.0 | 8,176 | 843 | 10.3 | 736 | 317 | 43.1 |
| Not Married | 2,904 | 434 | 14.9 | 2,904 | 395 | 13.6 | 434 | 164 | 37.8 |
| **Living Arrangement** |  |  |  |  |  |  |  |  |  |
| Living Alone | 1,688 | 235 | 13.9 | 1,688 | 232 | 13.7 | 235 | 81 | 34.5 |
| not Living Alone | 9,392 | 935 | 10.0 | 9,392 | 1,006 | 10.7 | 935 | 400 | 42.8 |
| **Educational Attainment** |  |  |  |  |  |  |  |  |  |
| <=9 years | 3,647 | 479 | 13.1 | 3,647 | 505 | 13.8 | 479 | 188 | 39.2 |
| >9 years | 7,433 | 691 | 9.3 | 7,433 | 733 | 9.9 | 691 | 293 | 42.4 |
| **Employment** |  |  |  |  |  |  |  |  |  |
| Employed | 2,927 | 130 | 4.4 | 2,927 | 191 | 6.5 | 130 | 53 | 40.8 |
| Not Employed | 8,153 | 1,040 | 12.8 | 8,153 | 1,047 | 12.8 | 1,040 | 428 | 41.2 |
| **Equivalent Income** |  |  |  |  |  |  |  |  |  |
| <2 millions/year | 2,901 | 422 | 14.5 | 2,901 | 388 | 13.4 | 422 | 168 | 39.8 |
| 2-4 millions/year | 4,607 | 456 | 9.9 | 4,607 | 523 | 11.4 | 456 | 198 | 43.4 |
| >4 millions/year | 3,572 | 292 | 8.2 | 3,572 | 327 | 9.2 | 292 | 115 | 39.4 |
| **BMI** |  |  |  |  |  |  |  |  |  |
| Underweight | 785 | 136 | 17.3 | 785 | 135 | 17.2 | 136 | 62 | 45.6 |
| Normalweight | 7,594 | 747 | 9.8 | 7,594 | 824 | 10.9 | 747 | 299 | 40.0 |
| Overweight | 2,701 | 287 | 10.6 | 2,701 | 279 | 10.3 | 287 | 120 | 41.8 |
| **Depressive Symptoms** |  |  |  |  |  |  |  |  |  |
| No Depression | 8,939 | 870 | 9.7 | 8,939 | 923 | 10.3 | 870 | 352 | 40.5 |
| Tendency toward Depression | 1,758 | 248 | 14.1 | 1,758 | 249 | 14.2 | 248 | 107 | 43.1 |
| Depression | 383 | 52 | 13.6 | 383 | 66 | 17.2 | 52 | 22 | 42.3 |
| **Dementia** |  |  |  |  |  |  |  |  |  |
| No | 11,051 | 1,157 | 10.5 | 11,051 | 1,230 | 11.1 | 1,157 | 476 | 41.1 |
| Yes | 29 | 13 | 44.8 | 29 | 8 | 27.6 | 13 | 5 | 38.5 |
| **Hypertension** |  |  |  |  |  |  |  |  |  |
| No | 6,116 | 617 | 10.1 | 6,116 | 658 | 10.8 | 617 | 251 | 40.7 |
| Yes | 4,964 | 553 | 11.1 | 4,964 | 580 | 11.7 | 553 | 230 | 41.6 |
| **Diabetes** |  |  |  |  |  |  |  |  |  |
| No | 9,700 | 1,003 | 10.3 | 9,700 | 1,053 | 10.9 | 1,003 | 408 | 40.7 |
| Yes | 1,380 | 167 | 12.1 | 1,380 | 185 | 13.4 | 167 | 73 | 43.7 |
| **Stroke** |  |  |  |  |  |  |  |  |  |
| No | 10,805 | 1,126 | 10.4 | 10,805 | 1,188 | 11.0 | 1,126 | 458 | 40.7 |
| Yes | 275 | 44 | 16.0 | 275 | 50 | 18.2 | 44 | 23 | 52.3 |
| **Cancer** |  |  |  |  |  |  |  |  |  |
| No | 10,656 | 1,113 | 10.4 | 10,656 | 1,135 | 10.7 | 1,113 | 454 | 40.8 |
| Yes | 424 | 57 | 13.4 | 424 | 103 | 24.3 | 57 | 27 | 47.4 |
| **Smoking** |  |  |  |  |  |  |  |  |  |
| Non smoker | 6,649 | 672 | 10.1 | 6,649 | 552 | 8.3 | 672 | 227 | 33.8 |
| Past Smoker | 3,274 | 373 | 11.4 | 3,274 | 517 | 15.8 | 373 | 202 | 54.2 |
| Current Smoker | 1,157 | 125 | 10.8 | 1,157 | 169 | 14.6 | 125 | 52 | 41.6 |
| **Drinking** |  |  |  |  |  |  |  |  |  |
| Non Drinker | 5,654 | 629 | 11.1 | 5,654 | 534 | 9.4 | 629 | 233 | 37.0 |
| Past Drinker | 1,177 | 164 | 13.9 | 1,177 | 224 | 19.0 | 164 | 79 | 48.2 |
| Current Drinker | 4,249 | 377 | 8.9 | 4,249 | 480 | 11.3 | 377 | 169 | 44.8 |
| **Walking Time** |  |  |  |  |  |  |  |  |  |
| >= 30 mins | 8,186 | 758 | 9.3 | 8,186 | 808 | 9.9 | 758 | 299 | 39.4 |
| <30 mins | 2,894 | 412 | 14.2 | 2,894 | 430 | 14.9 | 412 | 182 | 44.2 |

^a^Transition 2 in this table include those who experience disabled before death too.

**Supplementary Table 4.** Expected Life Expectancy at 65 years old (years) for Each Sex, after imputation (n=11,080)

| **Sex** | **Healthy Life Expectancy** | **HLE 95% CI (Lower - Higher)** | **Life Expectancy with Disability** | **LED 95% CI (Lower - Higher)** | **Total Life Expectancy** | **TLE 95% CI (Lower - Higher)** |
| --- | --- | --- | --- | --- | --- | --- |
| **Male** | 22.75 | 21.57 - 24.00 | 1.05 | 0.64 - 1.71 | 23.80 | 22.21 - 25.70 |
| **Female** | 24.25 | 23.22 - 25.32 | 2.08 | 1.44 - 3.01 | 26.33 | 24.66 - 28.33 |

Abbreviation: HLE = Healthy Life Expectancy; LED = Life Expectancy With Disability; TLE = Total Life Expectancy; CI = Confidence Interval

* Estimated from fully adjusted model using *Royston-Parmar flexible Parametric Model*

* Estimated model adjusted with the exposure (oral frailty, dental visits) and covariates *(age, sex, marital status, living arrangement, education level, employment status, household income, BMI, depressive symptoms, hypertension, diabetes, stroke, cancer, dementia, smoking status, alcohol consumption, walking time)* prevalence to show population average estimates.

**Supplementary Table 5.** Difference in Total Life Expectancy, Healthy Life Expectancy, and Life Expectancy with Disability at 65 years old with different Oral Frailty Status and Dental Visit (in years) for Each Sex, after imputation (n=11,080)

| **Category** | **Dental Visit** | **Healthy Life Expectancy** | **Differences in Healthy Life Expectancy** | **Life Expectancy with Disability** | **Differences in Life Expectancy with Disability** | **Total Life Expectancy** | **Differences in Total Life Expectancy** |
| --- | --- | --- | --- | --- | --- | --- | --- |
| **Male** | | | | | | | |
| **Non Frail** | With Visit | 23.85 | 0.97 | 0.98 | -0.03 | 24.8 | 0.94 |
|  | Without Visit | 22.88 | Ref | 1.01 | Ref | 23.9 | Ref |
| **Prefrail** | With Visit | 22.87 | 1.07 | 1.06 | -0.02 | 23.9 | 1.05 |
|  | Without Visit | 21.79 | Ref | 1.09 | Ref | 22.9 | Ref |
| **Frail** | With Visit | 22.50 | 1.12 | 1.09 | -0.02 | 23.6 | 1.10 |
|  | Without Visit | 21.38 | Ref | 1.11 | Ref | 22.5 | Ref |
| **Female** | | | | | | | |
| **Non Frail** | With Visit | 25.13 | 0.75 | 1.87 | -0.15 | 27.0 | 0.60 |
|  | Without Visit | 24.38 | Ref | 2.01 | Ref | 26.4 | Ref |
| **Prefrail** | With Visit | 24.32 | 0.84 | 2.10 | -0.14 | 26.4 | 0.70 |
|  | Without Visit | 23.47 | Ref | 2.24 | Ref | 25.7 | Ref |
| **Frail** | With Visit | 24.07 | 0.88 | 2.16 | -0.14 | 26.2 | 0.73 |
|  | Without Visit | 23.19 | Ref | 2.30 | Ref | 25.5 | Ref |

Abbreviation: HLE = Healthy Life Expectancy; LED = Life Expectancy With Disability; TLE = Total Life Expectancy; CI = Confidence Interval

*Estimated using *Royston-Parmar flexible Parametric Model*

* Estimated model adjusted with the covariates prevalence *(age, sex, marital status, living arrangement, education level, employment status, household income, BMI, depressive symptoms, hypertension, diabetes, stroke, cancer, dementia, smoking status, alcohol consumption, walking time) )* to show population average estimates.

**Supplementary Table *6*** Multistate Model: Hazard Ratio of Oral Frailty and Dental Visit with Disability or Death in Each Transition excluding a 1-year onset from baseline, after imputation (n=10,918)

| Oral Frailty Status | Model I | | | Model II | | | Model III | | |
| --- | --- | --- | --- | --- | --- | --- | --- | --- | --- |
|  | HR | 95% CI Lower | 95% CI Higher | HR | 95% CI Lower | 95% CI Higher | HR | 95% CI Lower | 95% CI Higher |
| **Transition I : Healthy to Disabled** |  |  |  |  |  |  |  |  |  |
| Non OF | Ref |  |  | Ref |  |  | Ref |  |  |
| Pre OF | 1.32** | 1.14 | 1.53 | 1.31** | 1.13 | 1.52 | 1.16** | 1.00 | 1.35 |
| Pre OF | 1.52** | 1.25 | 1.84 | 1.50** | 1.24 | 1.82 | 1.17 | 0.96 | 1.43 |
| Dental Visit |  |  |  | Ref |  |  | Ref |  |  |
| No Dental Visit |  |  |  | 1.18** | 1.05 | 1.34 | 1.14** | 1.01 | 1.28 |
| **Transition 2 : Healthy to Death** |  |  |  |  |  |  |  |  |  |
| Non OF | Ref |  |  | Ref |  |  | Ref |  |  |
| Pre OF | 1.34** | 1.11 | 1.61 | 1.33** | 1.10 | 1.60 | 1.17** | 0.97 | 1.41 |
| Pre OF | 1.70** | 1.34 | 2.16 | 1.68** | 1.33 | 2.13 | 1.30** | 1.01 | 1.67 |
| Dental Visit |  |  |  | Ref |  |  | Ref |  |  |
| No Dental Visit |  |  |  | 1.32** | 1.14 | 1.54 | 1.27** | 1.09 | 1.48 |
| **Transition 3 : Disabled to Dead** |  |  |  |  |  |  |  |  |  |
| Non OF | Ref |  |  | Ref |  |  | Ref |  |  |
| Pre OF | 1.07 | 0.84 | 1.35 | 1.06 | 0.84 | 1.35 | 1.07 | 0.84 | 1.36 |
| Pre OF | 1.11 | 0.83 | 1.50 | 1.11 | 0.82 | 1.49 | 1.11 | 0.82 | 1.51 |
| Dental Visit |  |  |  | Ref |  |  | Ref |  |  |
| No Dental Visit |  |  |  | 1.08 | 0.89 | 1.31 | 1.08 | 0.89 | 1.31 |

Abbreviation: HR = Hazard Ratio; CI = Confidence Interval

*Estimated using *Royston-Parmar flexible Parametric Model*

*Model I : Adjusted with Age and Sex

*Mode II : Adjusted with Dental Visit, Age, and Sex

*Model III : Adjusted with Dental Visit and All Covariates (age, sex, marital status, living arrangement, education level, employment status, household income, BMI, depressive symptoms, hypertension, diabetes, stroke, cancer, dementia, smoking status, alcohol consumption, walking time)

**Supplementary Table 7.** Difference in Total Life Expectancy, Healthy Life Expectancy, and Life Expectancy with Disability at 65 years old with different Oral Frailty Status (in years) excluding a 1-year onset from baseline, after imputation (n=10,918)

| Sex | Oral Frailty Status | **Healthy Life Expectancy** | **Differences in Healthy Life Expectancy** | **Life Expectancy with Disability** | **Differences in Life Expectancy with Disability** | **Total Life Expectancy** | **Differences in Total Life Expectancy** |
| --- | --- | --- | --- | --- | --- | --- | --- |
| Male | Non OF | 22.12 | 1.02 | 1.52 | -0.01 | 23.63 | 1.32 |
|  | Pre OF | 21.19 | 0.40 | 1.60 | 0.07 | 22.79 | 0.47 |
|  | Pre OF | 20.79 | Ref | 1.53 | Ref | 22.32 | Ref |
| Female | Non OF | 23.95 | 1.03 | 2.56 | -0.19 | 26.50 | 0.84 |
|  | Pre OF | 23.17 | 0.25 | 2.80 | 0.06 | 25.97 | 0.30 |
|  | Pre OF | 22.92 | Ref | 2.75 | Ref | 25.67 | Ref |

*Estimated using *Royston-Parmar flexible Parametric Model*

* Estimated model adjusted with the covariates prevalence *(age, sex, marital status, living arrangement, education level, employment status, household income, BMI, depressive symptoms, hypertension, diabetes, stroke, cancer, dementia, smoking status, alcohol consumption, walking time) )* to show population average estimates.

**Supplementary Table 8.** Difference in Total Life Expectancy, Healthy Life Expectancy, and Life Expectancy with Disability at 65 years old with different Oral Frailty Status and Dental Visit (in years) for Each Sex excluding a 1-year onset from baseline, after imputation (n=10,918)

| **Category** | **Dental Visit** | **Healthy Life Expectancy** | **Differences in Healthy Life Expectancy** | **Life Expectancy with Disability** | **Differences in Life Expectancy with Disability** | **Total Life Expectancy** | **Differences in Total Life Expectancy** |
| --- | --- | --- | --- | --- | --- | --- | --- |
| **Male** | | | | | | | |
| **Non OF** | With Visit | 22.65 | 1.11 | 1.50 | -0.02 | 24.15 | 1.09 |
|  | Without Visit | 21.54 | Ref | 1.52 | Ref | 23.07 | Ref |
| **Pre OF** | With Visit | 21.77 | 1.18 | 1.59 | 0.00 | 23.36 | 1.18 |
|  | Without Visit | 20.58 | Ref | 1.60 | Ref | 22.18 | Ref |
| **OF** | With Visit | 21.39 | 1.23 | 1.53 | 0.01 | 22.91 | 1.24 |
|  | Without Visit | 20.16 | Ref | 1.52 | Ref | 21.68 | Ref |
| **Female** | | | | | | | |
| **Non OF** | With Visit | 24.35 | 0.83 | 2.47 | -0.16 | 26.82 | 0.67 |
|  | Without Visit | 23.52 | Ref | 2.64 | Ref | 26.15 | Ref |
| **Pre OF** | With Visit | 23.61 | 0.90 | 2.72 | -0.16 | 26.33 | 0.75 |
|  | Without Visit | 22.70 | Ref | 2.88 | Ref | 25.58 | Ref |
| **OF** | With Visit | 23.38 | 0.94 | 2.67 | -0.14 | 26.05 | 0.80 |
|  | Without Visit | 22.43 | Ref | 2.82 | Ref | 25.25 | Ref |

*Estimated using *Royston-Parmar flexible Parametric Model*

* Estimated model adjusted with the covariates prevalence *(age, sex, marital status, living arrangement, education level, employment status, household income, BMI, depressive symptoms, hypertension, diabetes, stroke, cancer, dementia, smoking status, alcohol consumption, walking time) )* to show population average estimates.

**Supplementary Table 9**. Multistate Model: Hazard Ratio of Oral Frailty and Dental Visit with Disability or Death in Each Transition using Gompertz Distribution, after imputation (n=11,080)

| Oral Frailty Status | Model I | | | Model II | | | Model III | | |
| --- | --- | --- | --- | --- | --- | --- | --- | --- | --- |
|  | HR | 95% CI Lower | 95% CI Higher | HR | 95% CI Lower | 95% CI Higher | HR | 95% CI Lower | 95% CI Higher |
| **Transition I : Healthy to Disabled** |  |  |  |  |  |  |  |  |  |
| Non OF | Ref |  |  | Ref |  |  | Ref |  |  |
| Pre OF | 1.35** | 1.16 | 1.56 | 1.33** | 1.15 | 1.54 | 1.19** | 1.03 | 1.39 |
| Pre OF | 1.59** | 1.32 | 1.92 | 1.58** | 1.31 | 1.90 | 1.23** | 1.01 | 1.50 |
| Dental Visit |  |  |  | Ref |  |  | Ref |  |  |
| No Dental Visit |  |  |  | 1.20** | 1.06 | 1.35 | 1.16** | 1.03 | 1.30 |
| **Transition 2 : Healthy to Death** |  |  |  |  |  |  |  |  |  |
| Non OF | Ref |  |  | Ref |  |  | Ref |  |  |
| Pre OF | 1.40** | 1.18 | 1.68 | 1.39** | 1.17 | 1.66 | 1.22** | 1.02 | 1.46 |
| Pre OF | 1.75** | 1.40 | 2.20 | 1.73** | 1.38 | 2.18 | 1.34** | 1.05 | 1.71 |
| Dental Visit |  |  |  | Ref |  |  | Ref |  |  |
| No Dental Visit |  |  |  | 1.29** | 1.12 | 1.49 | 1.25** | 1.08 | 1.45 |
| **Transition 3 : Disabled to Dead** |  |  |  |  |  |  |  |  |  |
| Non OF | Ref |  |  | Ref |  |  | Ref |  |  |
| Pre OF | 1.06 | 0.84 | 1.33 | 1.06 | 0.84 | 1.33 | 1.05 | 0.83 | 1.33 |
| Pre OF | 1.05 | 0.79 | 1.40 | 1.04 | 0.78 | 1.39 | 1.04 | 0.77 | 1.39 |
| Dental Visit |  |  |  | Ref |  |  | Ref |  |  |
| No Dental Visit |  |  |  | 1.08 | 0.90 | 1.30 | 1.08 | 0.89 | 1.30 |

Abbreviation: HR = Hazard Ratio; CI = Confidence Interval

*Estimated using *Gompertz Distribution Model*

*Model I : Adjusted with Age and Sex

*Mode II : Adjusted with Dental Visit, Age, and Sex

*Model III : Adjusted with Dental Visit and All Covariates (age, sex, marital status, living arrangement,education level, employment status, household income, BMI, depressive symptoms, hypertension, diabetes, stroke, cancer, dementia, smoking status, alcohol consumption, walking time)

**Supplementary Table 10.** Difference in Total Life Expectancy, Healthy Life Expectancy, and Life Expectancy with Disability at 65 years old with different Oral Frailty Status (in years), using Gompertz distribution (n=11,080)

| Sex | Oral Frailty Status | **Healthy Life Expectancy** | **Differences in Healthy Life Expectancy** | **Life Expectancy with Disability** | **Differences in Life Expectancy with Disability** | **Total Life Expectancy** | **Differences in Total Life Expectancy** |
| --- | --- | --- | --- | --- | --- | --- | --- |
| Male | Non OF | 14.81 | 0.82 | 4.64 | 0.37 | 19.46 | 1.19 |
|  | Pre OF | 14.19 | 0.20 | 4.35 | 0.08 | 18.54 | 0.27 |
|  | Pre OF | 13.99 |  | 4.27 | Ref | 18.26 | Ref |
| Female | Non OF | 15.44 | 1.02 | 8.46 | 0.14 | 23.90 | 0.88 |
|  | Pre OF | 14.85 | 0.15 | 8.36 | 0.04 | 23.21 | 0.20 |
|  | Pre OF | 14.70 | Ref | 8.32 | Ref | 23.02 | Ref |

*Estimated using *Gompertz Distribution Model*

* Estimated model adjusted with the covariates prevalence *(age, sex, marital status, living arrangement, education level, employment status, household income, BMI, depressive symptoms, hypertension, diabetes, stroke, cancer, dementia, smoking status, alcohol consumption, walking time) )* to show population average estimates.

**Supplementary Table 11.** Difference in Total Life Expectancy, Healthy Life Expectancy, and Life Expectancy with Disability at 65 years old with different Oral Frailty Status and Dental Visit (in years) for Each Sex using Gompertz distribution, after imputation (n=11,080)

| **Category** | **Dental Visit** | **Healthy Life Expectancy** | **Differences in Healthy Life Expectancy** | **Life Expectancy with Disability** | **Differences in Life Expectancy with Disability** | **Total Life Expectancy** | **Differences in Total Life Expectancy** |
| --- | --- | --- | --- | --- | --- | --- | --- |
| **Male** | | | | | | | |
| **Non OF** | With Visit | 15.10 | 0.59 | 4.84 | 0.41 | 19.95 | 1.00 |
|  | Without Visit | 14.52 | Ref | 4.43 | Ref | 18.95 | Ref |
| **Pre OF** | With Visit | 14.48 | 0.60 | 4.48 | 0.35 | 18.96 | 0.94 |
|  | Without Visit | 13.88 | Ref | 4.13 | Ref | 18.02 | Ref |
| **OF** | With Visit | 14.29 | 0.60 | 4.49 | 0.44 | 18.78 | 1.04 |
|  | Without Visit | 13.68 | Ref | 4.05 | Ref | 17.73 | Ref |
| **Female** | | | | | | | |
| **Non OF** | With Visit | 15.70 | 0.52 | 8.56 | 0.22 | 24.26 | 0.74 |
|  | Without Visit | 15.17 | Ref | 8.34 | Ref | 23.51 | Ref |
| **Pre OF** | With Visit | 15.11 | 0.53 | 8.49 | 0.27 | 23.60 | 0.80 |
|  | Without Visit | 14.59 | Ref | 8.21 | Ref | 22.80 | Ref |
| **OF** | With Visit | 14.96 | 0.53 | 8.45 | 0.29 | 23.41 | 0.82 |
|  | Without Visit | 14.43 | Ref | 8.16 | Ref | 22.60 | Ref |

*Estimated using *Gompertz Distribution Model*

* Estimated model adjusted with the covariates prevalence *(age, sex, marital status, living arrangement, education level, employment status, household income, BMI, depressive symptoms, hypertension, diabetes, stroke, cancer, dementia, smoking status, alcohol consumption, walking time) )* to show population average estimates.

**Supplementary Table 12.** Multistate Model: Hazard Ratio of Oral Frailty and Dental Visit with Disability or Death in Each Transition using Weibull Distribution, after imputation (n=11,080)

| Oral Frailty Status | Model I | | | Model II | | | Model III | | |
| --- | --- | --- | --- | --- | --- | --- | --- | --- | --- |
|  | HR | 95% CI Lower | 95% CI Higher | HR | 95% CI Lower | 95% CI Higher | HR | 95% CI Lower | 95% CI Higher |
| **Transition I : Healthy to Disabled** |  |  |  |  |  |  |  |  |  |
| Non OF | Ref |  |  | Ref |  |  | Ref |  |  |
| Pre OF | 1.34** | 1.16 | 1.56 | 1.33** | 1.15 | 1.54 | 1.19** | 1.03 | 1.39 |
| Pre OF | 1.59** | 1.32 | 1.92 | 1.58** | 1.31 | 1.90 | 1.23** | 1.01 | 1.50 |
| Dental Visit |  |  |  | Ref |  |  | Ref |  |  |
| No Dental Visit |  |  |  | 1.20** | 1.06 | 1.35 | 1.16** | 1.03 | 1.30 |
| **Transition 2 : Healthy to Death** |  |  |  |  |  |  |  |  |  |
| Non OF | Ref |  |  | Ref |  |  | Ref |  |  |
| Pre OF | 1.40** | 1.18 | 1.68 | 1.39** | 1.17 | 1.66 | 1.22** | 1.02 | 1.46 |
| Pre OF | 1.75** | 1.40 | 2.20 | 1.73** | 1.38 | 2.18 | 1.34** | 1.05 | 1.71 |
| Dental Visit |  |  |  | Ref |  |  | Ref |  |  |
| No Dental Visit |  |  |  | 1.29** | 1.12 | 1.49 | 1.25** | 1.08 | 1.45 |
| **Transition 3 : Disabled to Dead** |  |  |  |  |  |  |  |  |  |
| Non OF | Ref |  |  | Ref |  |  | Ref |  |  |
| Pre OF | 1.06 | 0.84 | 1.33 | 1.06 | 0.84 | 1.33 | 1.05 | 0.83 | 1.33 |
| Pre OF | 1.05 | 0.79 | 1.40 | 1.04 | 0.78 | 1.39 | 1.04 | 0.77 | 1.39 |
| Dental Visit |  |  |  | Ref |  |  | Ref |  |  |
| No Dental Visit |  |  |  | 1.08 | 0.90 | 1.30 | 1.08 | 0.89 | 1.30 |

Abbreviation: HR = Hazard Ratio; CI = Confidence Interval

*Estimated using *Weibull Distribution Model*

*Model I : Adjusted with Age and Sex

*Mode II : Adjusted with Dental Visit, Age, and Sex

*Model III : Adjusted with Dental Visit and All Covariates (age, sex, marital status, living arrangement, education level, employment status, household income, BMI, depressive symptoms, hypertension, diabetes, stroke, cancer, dementia, smoking status, alcohol consumption, walking time)

**Supplementary Table 13.** Difference in Total Life Expectancy, Healthy Life Expectancy, and Life Expectancy with Disability at 65 years old with different Sex and Oral Frailty Status (in years) using Weibull Distribution, after imputation (n=11,080)

| Sex | Oral Frailty Status | **Healthy Life Expectancy** | **Differences in Healthy Life Expectancy** | **Life Expectancy with Disability** | **Differences in Life Expectancy with Disability** | **Total Life Expectancy** | **Differences in Total Life Expectancy** |
| --- | --- | --- | --- | --- | --- | --- | --- |
| Male | Non OF | 24.18 | 1.30 | 0.91 | -0.10 | 25.09 | 1.19 |
|  | Pre OF | 23.23 | 0.35 | 0.99 | -0.02 | 24.22 | 0.32 |
|  | Pre OF | 22.88 | Ref | 1.01 | Ref | 23.89 | Ref |
| Female | Non OF | 25.37 | 1.02 | 1.75 | -0.27 | 27.12 | 0.75 |
|  | Pre OF | 24.59 | 0.24 | 1.97 | -0.06 | 26.56 | 0.18 |
|  | Pre OF | 24.35 | Ref | 2.02 | Ref | 26.37 | Ref |

*Estimated using *Weibull Distribution Model*

* Estimated model adjusted with the covariates prevalence *(age, sex, marital status, living arrangement, education level, employment status, household income, BMI, depressive symptoms, hypertension, diabetes, stroke, cancer, dementia, smoking status, alcohol consumption, walking time) )* to show population average estimates.

**Supplementary Table 14**. Difference in Total Life Expectancy, Healthy Life Expectancy, and Life Expectancy with Disability at 65 years old with different Oral Frailty Status and Dental Visit (in years) for Each Sex using Weibull distribution, after imputation (n=11,080)

| **Category** | **Dental Visit** | **Healthy Life Expectancy** | **Differences in Healthy Life Expectancy** | **Life Expectancy with Disability** | **Differences in Life Expectancy with Disability** | **Total Life Expectancy** | **Differences in Total Life Expectancy** |
| --- | --- | --- | --- | --- | --- | --- | --- |
| **Male** | | | | | | | |
| **Non OF** | With Visit | 24.60 | 0.87 | 0.89 | -0.04 | 25.49 | 0.84 |
|  | Without Visit | 23.73 | Ref | 0.92 | Ref | 24.65 | Ref |
| **Pre OF** | With Visit | 23.70 | 0.98 | 0.97 | -0.03 | 24.67 | 0.95 |
|  | Without Visit | 22.72 | Ref | 1.00 | Ref | 23.72 | Ref |
| **OF** | With Visit | 23.38 | 1.03 | 0.99 | -0.03 | 24.37 | 1.00 |
|  | Without Visit | 22.35 | Ref | 1.02 | Ref | 23.37 | Ref |
| **Female** | | | | | | | |
| **Non OF** | With Visit | 25.69 | 0.67 | 1.68 | -0.14 | 27.37 | 0.53 |
|  | Without Visit | 25.02 | Ref | 1.82 | Ref | 26.84 | Ref |
| **Pre OF** | With Visit | 24.96 | 0.76 | 1.90 | -0.14 | 26.85 | 0.62 |
|  | Without Visit | 24.19 | Ref | 2.04 | Ref | 26.23 | Ref |
| **OF** | With Visit | 24.73 | 0.80 | 1.95 | -0.14 | 26.68 | 0.65 |
|  | Without Visit | 23.94 | Ref | 2.09 | Ref | 26.03 | Ref |

*Estimated using *Weibull Distribution Model*

* Estimated model adjusted with the covariates prevalence *(age, sex, marital status, living arrangement, education level, employment status, household income, BMI, depressive symptoms, hypertension, diabetes, stroke, cancer, dementia, smoking status, alcohol consumption, walking time) )* to show population average estimates.

**Supplementary Table 15.** Multistate Model: Hazard Ratio of Oral Frailty and Dental Visit with Disability or Death in Each Transition, in Complete Case Analysis (n=5,890)

| Oral Frailty Status | Model I | | | Model II | | | Model III | | |
| --- | --- | --- | --- | --- | --- | --- | --- | --- | --- |
|  | HR | 95% CI Lower | 95% CI Higher | HR | 95% CI Lower | 95% CI Higher | HR | 95% CI Lower | 95% CI Higher |
| **Transition I : Healthy to Disabled** |  |  |  |  |  |  |  |  |  |
| Non Frail | Ref |  |  | Ref |  |  | Ref |  |  |
| Pre Frail | 1.55 | 1.24 | 1.95 | 1.54 | 1.22 | 1.94 | 1.35 | 1.06 | 1.7 |
| Frail | 2.21 | 1.66 | 2.94 | 2.19 | 1.65 | 2.92 | 1.55 | 1.15 | 2.09 |
| Dental Visit |  |  |  | Ref |  |  | Ref |  |  |
| No Dental Visit |  |  |  | 1.25 | 1.04 | 1.5 | 1.17 | 0.97 | 1.41 |
| **Transition 2 : Healthy to Death** |  |  |  |  |  |  |  |  |  |
| Non Frail | Ref |  |  | Ref |  |  | Ref |  |  |
| Pre Frail | 1.72 | 1.35 | 2.2 | 1.71 | 1.34 | 2.19 | 1.44 | 1.12 | 1.86 |
| Frail | 1.92 | 1.38 | 2.67 | 1.91 | 1.38 | 2.66 | 1.42 | 1.01 | 2 |
| Dental Visit |  |  |  | Ref |  |  | Ref |  |  |
| No Dental Visit |  |  |  | 1.24 | 1.02 | 1.52 | 1.16 | 0.95 | 1.42 |
| **Transition 3 : Disabled to Dead** |  |  |  |  |  |  |  |  |  |
| Non Frail | Ref |  |  | Ref |  |  | Ref |  |  |
| Pre Frail | 0.92 | 0.65 | 1.29 | 0.92 | 0.65 | 1.29 | 0.93 | 0.64 | 1.33 |
| Frail | 0.92 | 0.61 | 1.4 | 0.91 | 0.6 | 1.39 | 0.95 | 0.6 | 1.51 |
| Dental Visit |  |  |  | Ref |  |  | Ref |  |  |
| No Dental Visit |  |  |  | 0.95 | 0.72 | 1.24 | 0.88 | 0.65 | 1.17 |

Abbreviation: HR = Hazard Ratio; CI = Confidence Interval

*Estimated using *Royston-Parmar flexible Parametric Model*

*Model I : Adjusted with Age and Sex

*Mode II : Adjusted with Dental Visit, Age, and Sex

*Model III : Adjusted with Dental Visit and All Covariates (age, sex, marital status, living arrangement, education level, employment status, household income, BMI, depressive symptoms, hypertension, diabetes, stroke, cancer, dementia, smoking status, alcohol consumption, walking time)

**Supplementary Table 16.** Difference in Total Life Expectancy, Healthy Life Expectancy, and Life Expectancy with Disability at 65 years old with different Oral Frailty Status (years) in Complete Case Analysis (n=5,890)

| Sex | Oral Frailty Status | **Healthy Life Expectancy** | **Differences in Healthy Life Expectancy** | **Life Expectancy with Disability** | **Differences in Life Expectancy with Disability** | **Total Life Expectancy** | **Differences in Total Life Expectancy** |
| --- | --- | --- | --- | --- | --- | --- | --- |
| Male | Non OF | 18.15 | 2.84 | 0.95 | -0.21 | 19.1 | 2.63 |
|  | Pre OF | 15.7 | 0.39 | 1.08 | -0.08 | 16.78 | 0.31 |
|  | Pre OF | 15.31 | Ref | 1.16 | Ref | 16.47 | Ref |
| Female | Non OF | 19.95 | 1.02 | 2.89 | -0.88 | 22.83 | 1.96 |
|  | OF | 17.65 | 0.55 | 3.47 | -0.29 | 21.12 | 0.25 |
|  | Pre OF | 17.11 | Ref | 3.76 | Ref | 20.87 | Ref |

*Estimated using *Royston-Parmar flexible Parametric Model*

* Estimated model adjusted with the covariates prevalence *(age, sex, marital status, living arrangement, education level, employment status, household income, BMI, depressive symptoms, hypertension, diabetes, stroke, cancer, dementia, smoking status, alcohol consumption, walking time) )* to show population average estimates.

**Supplementary Table 17.** Difference in Total Life Expectancy, Healthy Life Expectancy, and Life Expectancy with Disability at 65 years old with different Oral Frailty Status and Dental Visit in Complete Case Analysis (n=5,890*)*

| **Category** | **Dental Visit** | **Healthy Life Expectancy** | **Differences in Healthy Life Expectancy** | **Life Expectancy with Disability** | **Differences in Life Expectancy with Disability** | **Total Life Expectancy** | **Differences in Total Life Expectancy** |
| --- | --- | --- | --- | --- | --- | --- | --- |
| **Male** | | | | | | | |
| **Non OF** | With Visit | 19.71 | 1.07 | 1.09 | -0.20 | 20.81 | 0.87 |
|  | Without Visit | 18.64 | Ref | 1.29 | Ref | 19.93 | Ref |
| **Pre OF** | With Visit | 17.21 | 1.13 | 1.29 | -0.20 | 18.50 | 0.92 |
|  | Without Visit | 16.08 | Ref | 1.49 | Ref | 17.57 | Ref |
| **OF** | With Visit | 17.03 | 1.13 | 1.43 | -0.23 | 18.46 | 0.90 |
|  | Without Visit | 15.90 | Ref | 1.66 | Ref | 17.56 | Ref |
| **Female** | | | | | | | |
| **Non OF** | With Visit | 22.07 | 1.02 | 2.25 | -0.34 | 24.32 | 0.60 |
|  | Without Visit | 21.12 | Ref | 2.59 | Ref | 23.71 | Ref |
| **Pre OF** | With Visit | 19.88 | 1.02 | 2.76 | -0.37 | 22.64 | 0.69 |
|  | Without Visit | 18.81 | Ref | 3.13 | Ref | 21.94 | Ref |
| **OF** | With Visit | 19.58 | 1.02 | 3.09 | -0.42 | 22.68 | 0.66 |
|  | Without Visit | 18.50 | Ref | 3.51 | Ref | 22.01 | Ref |

*Estimated using *Royston-Parmar flexible Parametric Model*

* Estimated model adjusted with dental visit and all covariates *(age, sex, marital status, living arrangement, education level, employment status, household income, BMI, depressive symptoms, hypertension, diabetes, stroke, cancer, dementia, smoking status, alcohol consumption, walking time)*

**Supplementary Table 18.** Multistate Model: Hazard Ratio of Oral Frailty and Dental Visit with Disability or Death in Each Transition, Comparison of Hazard Ratios (HR) for Different Cutoff Points of Oral Frailty (n=11,080*)*

| Oral Frailty Status | **Cut Off 1 (3 Category)** | | | **Cut Off 2 (3 Category)** | | | **Cut Off (3 Category)** | | | **Cut Off 4 (2 Category)** | | |
| --- | --- | --- | --- | --- | --- | --- | --- | --- | --- | --- | --- | --- |
|  | HR | 95% CI Lower | 95% CI Higher | HR | 95% CI Lower | 95% CI Higher | HR | 95% CI Lower | 95% CI Higher | HR | 95% CI Lower | 95% CI Higher |
| **Transition I : Healthy to Disabled** | | | | | | | | | | | | |
| Non OF | Ref |  |  | Ref |  |  | Ref |  |  | Ref |  |  |
| Pre OF | **1.19**** | 1.03 | 1.38 | **1.20**** | 1.02 | 1.41 | 1.07 | 0.94 | 1.21 |  |  |  |
| OF | **1.23**** | 1.01 | 1.50 | **1.20**** | 1.02 | 1.41 | 1.04 | 0.79 | 1.36 | 1.03 | 0.96 | 1.10 |
| Dental Visit | Ref |  |  | Ref |  |  | Ref |  |  |  |  |  |
| No Dental Visit | **1.16**** | 1.03 | 1.30 | **1.16**** | 1.03 | 1.30 | **1.16**** | 1.03 | 1.31 | **1.16**** | 1.03 | 1.31 |
| **Transition 2 : Healthy to Death** | | | | | | | | | | | | |
| Non OF | Ref |  |  | Ref |  |  | Ref |  |  | Ref |  |  |
| Pre OF | **1.21**** | 1.02 | 1.46 | **1.24**** | 1.02 | 1.51 | 1.08 | 0.92 | 1.26 |  |  |  |
| OF | **1.34**** | 1.05 | 1.71 | **1.24**** | 1.02 | 1.51 | 1.10 | 0.78 | 1.54 | 1.08 | 0.93 | 1.26 |
| Dental Visit | Ref |  |  | Ref |  |  | Ref |  |  |  |  |  |
| No Dental Visit | **1.25**** | 1.08 | 1.45 | **1.25**** | 1.08 | 1.45 | **1.25**** | 1.08 | 1.45 | **1.25**** | 1.08 | 1.45 |
| **Transition 3 : Disabled to Dead** | | | | | | | | | | | | |
| Non OF | Ref |  |  | Ref |  |  | Ref |  |  | Ref |  |  |
| Pre OF | 1.05 | 0.83 | 1.33 | 1.00 | 0.77 | 1.29 | 1.14 | 0.94 | 1.38 |  |  |  |
| OF | 1.03 | 0.77 | 1.38 | 1.08 | 0.85 | 1.38 | 0.71 | 0.45 | 1.11 | 1.08 | 0.90 | 1.31 |
| Dental Visit | Ref |  |  | Ref |  |  | Ref |  |  |  |  |  |
| No Dental Visit | 1.08 | 0.89 | 1.30 | 1.08 | 0.89 | 1.30 | 1.08 | 0.90 | 1.31 | 1.08 | 0.90 | 1.31 |

**Cut Off 1**: Non Frail (OF score = 0; n = 3,756), Prefrail (OF score = 1-2; n = 5,997), Frail (OF score = 3-5; n = 3,520)

**Cut Off 2**: Non Frail (OF score = 0; n = 3,756), Prefrail (OF score = 1; n = 3,804), Frail (OF score = 2-5; n = 3,520)

**Cut Off 3**: Non Frail (OF score = 0-1; n = 7,560), Prefrail (OF score = 2-3; n = 3,142), Frail (OF score = 4-5; n = 378)
 **Cut Off 4**: Non Frail (OF score = 0-1; n = 7,560), Frail (OF score = 2-5; n = 3,520); *current consensus of Oral Frailty*

Adjusted with All Covariates (age, sex, marital status, living arrangement, education level, employment status, household income, BMI, depressive symptoms, hypertension, diabetes, stroke, cancer, dementia, smoking status, alcohol consumption, walking time)
